# Rebound growth of BRAF mutant pediatric glioma cells after MAPKi withdrawal is associated with MAPK reactivation and secretion of microglia-recruiting cytokines

**DOI:** 10.1007/s11060-024-04672-9

**Published:** 2024-04-17

**Authors:** Daniela Kocher, Lei Cao, Romain Guiho, Melanie Langhammer, Yun-Lu Lai, Pauline Becker, Hiba Hamdi, Dennis Friedel, Florian Selt, David Vonhören, Julia Zaman, Gintvile Valinciute, Sonja Herter, Daniel Picard, Johanna Rettenmeier, Kendra K. Maass, Kristian W. Pajtler, Marc Remke, Andreas von Deimling, Stefan Pusch, Stefan M. Pfister, Ina Oehme, David T.W. Jones, Sebastian Halbach, Tilman Brummer, Juan Pedro Martinez-Barbera, Olaf Witt, Till Milde, Romain Sigaud

**Affiliations:** 1 Hopp Children’s Cancer Center Heidelberg (KiTZ), Heidelberg, Germany; 2 National Center for Tumor Diseases (NCT), NCT Heidelberg, a partnership between DKFZ and Heidelberg University Hospital, Heidelberg, Germany; 3 German Cancer Research Center (DKFZ) Heidelberg, Clinical Cooperation Unit Pediatric Oncology, Heidelberg, Germany; 4 Faculty of Biosciences, Heidelberg University, Heidelberg, Germany; 5 Developmental Biology and Cancer Programme, Birth Defects Research Centre, Great Ormond Street Institute of Child Health, University College London, 30 Guilford Street, WC1N 1EH London, UK; 6 Department of Neurosurgery, Beijing Tiantan Hospital, Capital Medical University, Beijing, China; 7 Nantes Université, Oniris, INSERM, Regenerative Medicine and Skeleton, RMeS, UMR 1229, F-44000 Nantes, France; 8 Institute of Molecular Medicine and Cell Research (IMMZ), Faculty of Medicine, University of Freiburg, Freiburg, Germany; 9 Faculty of Biology, University of Freiburg, Freiburg, Germany; 10 Faculty of Medicine, Heidelberg University, Heidelberg, Germany; 11 Department of Neuropathology, Heidelberg University Hospital, Heidelberg, Germany; 12 German Cancer Research Center (DKFZ), Clinical Cooperation Unit Neuropathology, Heidelberg, Germany; 13 KiTZ Clinical Trial Unit (ZIPO), Department of Pediatric Hematology and Oncology, Heidelberg University Hospital, Heidelberg, Germany; 14 Department of Pediatric Oncology, Hematology, and Clinical Immunology, Medical Faculty, and University Hospital Düsseldorf, Heinrich Heine University, Düsseldorf, Germany; 15 German Cancer Consortium (DKTK), Partner site Essen/Düsseldorf, Düsseldorf, Germany; 16 Institute of Neuropathology, Medical Faculty and University Hospital Düsseldorf, Heinrich Heine University, Düsseldorf, Germany; 17 German Cancer Research Center (DKFZ), Division of Pediatric Neurooncology, Heidelberg, Germany; 18 Department of Pediatric Oncology, Hematology, Immunology and Pulmonology, Heidelberg University Hospital, Heidelberg, Germany; 19 Pediatric Hematology and Oncology, University Children’s Hospital, Saarland University, Homburg, Germany; 20 German Cancer Research Center (DKFZ) Heidelberg, Division of Pediatric Glioma Research, Heidelberg, Germany; 21 German Consortium for Translational Cancer Research (DKTK), Freiburg, Germany, German Cancer Research Center (DKFZ), Heidelberg, Germany; 22 German Cancer Research Center (DKFZ), Heidelberg, Germany; 23 Centre for Biological Signaling Studies BIOSS, University of Freiburg, Freiburg, Germany

**Keywords:** Pediatric low-grade glioma, MAPK inhibitor, Rebound growth, Treatment withdrawal, Cytokines, Tumor microenvironment

## Abstract

**Introduction:**

Patients with pediatric low-grade gliomas (pLGGs), the most common primary brain tumors in children, can often benefit from MAPK inhibitor (MAPKi) treatment. However, rapid tumor regrowth, also referred to as rebound growth, may occur once treatment is stopped, constituting a significant clinical challenge.

**Methods:**

Four patient-derived pediatric glioma models were investigated to model rebound growth in vitro based on viable cell counts in response to MAPKi treatment and withdrawal. A multi-omics dataset (RNA sequencing and LC-MS/MS based phospho-/proteomics) was generated to investigate possible rebound-driving mechanisms. Following in vitro validation, putative rebound-driving mechanisms were validated in vivo using the BT-40 orthotopic xenograft model.

**Results:**

Of the tested models, only a *BRAF*^V600E^-driven model (BT-40, with additional *CDKN2A/B*del) showed rebound growth upon MAPKi withdrawal. Using this model, we identified a rapid reactivation of the MAPK pathway upon MAPKi withdrawal in vitro, also confirmed in vivo. Furthermore, transient overactivation of key MAPK molecules at transcriptional (e.g. *FOS*) and phosphorylation (e.g. pMEK) levels, was observed in vitro. Additionally, we detected increased expression and secretion of cytokines (CCL2, CX3CL1, CXCL10 and CCL7) upon MAPKi treatment, maintained during early withdrawal. While increased cytokine expression did not have tumor cell intrinsic effects, presence of these cytokines in conditioned media led to increased attraction of microglia cells in vitro.

**Conclusion:**

Taken together, these data indicate rapid MAPK reactivation upon MAPKi withdrawal as a tumor cell intrinsic rebound-driving mechanism. Furthermore, increased secretion of microglia-recruiting cytokines may play a role in treatment response and rebound growth upon withdrawal, warranting further evaluation.

**Supplementary Information:**

The online version contains supplementary material available at 10.1007/s11060-024-04672-9.

## Introduction

Pediatric low-grade gliomas (pLGG) are the most common primary brain tumors in children [1]. They are a diverse group of WHO grade 1 and grade 2 glial and glioneuronal tumors [2], which typically contain a high proportion of tumor microenvironment (TME) cells, especially microglia/macrophages (30–50%) [3]. While pLGG patients have a good overall prognosis (15-year overall survival 80–90%), progression-free survival is considerably lower (15-year PFS: 55%), in particular in cases of incomplete resection (15-year PFS for non-resected patients: 27%) [4]. Incompletely resected pLGGs are a chronic disease, often requiring multiple lines of therapy, leading to accumulation of therapy-related sequelae [4]. This underscores the need for better and definitive therapies, to improve the quality of life for these patients.

pLGGs are almost exclusively driven by mutually exclusive alterations in different components of the extracellular-regulated kinase/mitogen-activated protein kinase (MAPK) pathway [5]. The most common alterations in pLGGs affect the *BRAF* gene, and comprise either structural *BRAF* rearrangements (~ 50% *KIAA1549:BRAF* fusions) or *BRAF* point mutations (~ 10% *BRAF*^V600E^ mutations) [3].

The fact that pLGGs are mainly driven by increased MAPK activation offers the possibility for targeted therapy using different small molecule inhibitors of the MAPK pathway. Several clinical trials investigating the efficacy of these inhibitors have shown promising effects [6]. Two studies assessing the efficacy of the BRAF type I ½ inhibitor [7] dabrafenib, one retrospective analysis [8] and one phase I/II clinical trial [9], showed an objective response rate (ORR) of 80% [8] and 44% [9] respectively. Furthermore, the combination of dabrafenib and trametinib was recently FDA-approved for the first-line treatment of *BRAF*^V600E^-driven pLGGs [10], as it showed an improved overall response (OR, defined as best overall complete or partial response; 47%), compared to standard-of-care (SOC) chemotherapy (OR: 11%), as well as less toxicity [11].

However, despite the efficacy of MAPKi treatment, a subset of patients experiences fast tumor regrowth upon treatment stop [8, 12]. This rapid tumor regrowth is also referred to as rebound growth, and constitutes a significant clinical challenge. Rebound growth is characterized by a rapid tumor regrowth (≥25%) within three months after treatment stop (Patricia O’Hare et al., Neuro Oncol., under review; e.g. median time to progression after dabrafenib treatment: 2.3 months) [8], and in some patients faster tumor growth is observed after MAPKi treatment compared to before treatment (clinical observation). While tumor regrowth is also observed in patients after chemotherapy treatment, time to progression is considered to be shorter in bona fide rebound (Patricia O’Hare et al., Neuro Oncol., under review). Interestingly, tumors showing rebound growth remain sensitive to the initial MAPKi treatment [8, 13], suggesting that acquired resistance mechanisms are not responsible for the observed pattern.

In the present study, we show that, in a *BRAF*^V600E^-driven model with co-occurring *CDKN2A/B* deletion, rebound growth after MAPKi withdrawal is associated with a fast reactivation of the MAPK pathway. Additionally, our results indicate a possible involvement of MAPKi-induced cytokine expression on the TME, specifically microglia cells.

## Materials and methods

### Cell culture

BT-40 cells, a kind gift from Prof. Peter Houghton, were culture in RPMI containing L-glutamine (cat. no 21,875,034, ThermoFisher Scientific) and 10% FCS (cat. no. F7524, Sigma Aldrich). DKFZ-BT66, DKFZ-BT308 and DKFZ-BT314 were cultured as described previously [14, 15] and used in proliferating and senescent state. As described previously, to culture cells in proliferating state oncogene-induced senescence, otherwise observed in these cells, is inhibited through the induction of the SV40-TAg [14, 15]. Further details on this system and the pipeline used to establish these models are described in detail in the original publications [14–16].

The microglia cell line HMC3 was purchased from the ATCC and cultured in MEM with L-glutamine (cat. no. 31,095,029) supplemented with 10% FCS, non-essential amino acids (cat. no. 11,140,035, ThermoFisher Scientific) and sodium pyruvate (cat. no. 11,360,039, ThermoFisher Scientific).

If not otherwise indicated, BT-40, DKFZ-BT308 and DKFZ-BT314 were seeded for experiments two days prior to experiment start in complete media (containing all supplements) and upon experiment start switched to minimum media (MM).

For further details see Supplementary materials and methods.

### Drug treatments and withdrawal

All inhibitors used for in vitro experiments are listed in Table S3. For details on drug concentration choice and the withdrawal procedure see Supplementary materials and methods.

### Metabolic activity assay for IC50 determination

Metabolic activity was measured using CellTiter-Glo 2.0 (cat. no. G9241, Promega) according to the manufacturer’s instructions. For further details see Supplementary materials and methods.

### Cell counting for growth curve analysis

Cells were counted using the Vi-CELL XR (Beckman Coulter; Software v2.03) using the settings described in Table S1. For further details see Supplementary materials and methods.

### Cell cycle analysis by flow cytometry

Cell cycle analysis by flow cytometry was performed as described previously [17].

### RNA isolation, cDNA synthesis and quantitative reverse transcription real-time PCR (RT-qPCR)

qPCR was performed as described previously [18]. Primers were purchased from Qiagen or Invitrogen (Table S5). For further details see Supplementary materials and methods.

### Protein extraction and immunoblotting

Antibodies used are listed in Table S6. For further details see Supplementary materials and methods.

### RNA sequencing and data processing

2 × 100 bp paired-end sequencing was performed on the Illumina NovaSeq 6000 according to the manufacturer’s protocol. For details see Supplementary materials and methods.

### LC-MS/MS proteomics and phosphoproteomics data generation and processing

Briefly, proteins were cleaved using Trypsin/Lys-C mix (cat. no. V5072, Promega) and either cleaned up using self-made SDB-PRS stage tips in case of proteomics or enriched for phospho-peptides using the High-Select™ TiO2 Phosphopeptide Enrichment Kit (cat. no. A32993, ThermoFisher Scientific) in case of phospho-proteomics analysis. For details on sample processing, data generation and processing see Supplementary materials and methods.

### Luminex-based multiplex assay

The Luminex-based multiplex assay was performed using the Bio-Plex 200 System (Bio-Rad) with the Bio-Plex Pro Reagent Kit 3 (cat. no. 171,304,090 M, Bio-Rad) using the Bio-Plex Pro HuCSP standard (cat. no. 12,007,919, Bio-Rad) and the Bio-Plex Pro Human Chemokine Standards (cat. no. 171DK0001, Bio-Rad) according to the manufacturer’s instructions. Custom detection antibody multiplexes were used as indicated in Table S7. For details see Supplementary materials and methods.

### Kinase phosphorylation array

Proteome Profiler Human Phospho-Kinase Array Kit (ARY003C, R&D systems) was used according to the manufacturer’s instructions using 400 µg of protein lysate per array set. Arrays were visualized with Amersham ECL Prime Western Blotting Detection Reagent (cat. no. RPN2232, GE Healthcare Dharmacon) using the Azure c400 imaging system (Azure Biosystems). Quantification was done using ImageJ (v2.9.0).

### Stimulation with recombinant cytokines

Cells were treated for 1 h with 100 ng/ml of recombinant human CCL2 (cat. no. 300-04, PeproTech), CX3CL1 (cat. no. 300 − 31, PeproTech), CXCL10 (cat. no. 300 − 12, PeproTech), CCL7 (cat. no. 300 − 17, PeproTech) or the combination of all four.

### Treatment with neutralizing antibodies

For cell count experiments and western blot analysis, cells were treated with antibodies neutralizing CCL2 (0.5 µg/ml; cat. no. MAB279, R&D systems), CX3CL1 (0.25 µg/ml; cat. no. MAB3652, R&D systems), CXCL10 (0.25 µg/mL; cat. no. MAB266, R&D systems) and CCL7 (0.1ng/ml; cat. no. MAB282, R&D systems) or mouse IgG (cat. no. MAB002, R&D systems).

### Conditioned media collection for transwell assay

For conditioned media collection, BT-40 were treated for five days, with media changes on day 2 and day 4 of treatment, and on day 5 treatment withdrawal was performed. CM (containing 2% FCS) was collected after five days of treatment (24 h after the last media change), and 24 h after treatment withdrawal. After collection, CM was centrifuged for 5 min at 1200 rpm and filtered using a 0.22 µM sterile filter (cat. no. SLGS033, Sigma Aldrich).

### Transwell migration assay

Transwell migration assays were performed using a 24-well transwell chamber (insert: 8 µM pore size; cat. no. 353,097, Corning; companion plate: 353,504, Corning). Detailed experimental procedures are described in the Supplementary materials and methods.

### Transwell co-culture

HMC3 cells were switched to RPMI containing 10% FCS upon seeding in the upper transwell chamber (6-well format, 0.4 µM pore size; cat. no. 353,493, Corning). Two days after seeding, transwell inserts were added into wells containing BT-40 (6-well companion plate, cat. no. 353,502, Corning) and media was switched to MM (RPMI, 2% FCS) for the whole transwell chamber.

### BT40 xenograft in vivo model

All mouse procedures were performed under license (PP8308129), following UK Home Office Animals (Scientific Procedures) Act 1986 and local institutional guidelines (UCL ethical review committee) and ARRIVE guidelines. For details on experimental procedures see Supplementary materials and methods.

### Statistics

Statistical analysis was either performed using GraphPad Prism (v8.0.2) or R Studio (R version 4.2.2). Statistical tests performed and number of independent biological replicates for each experiment are specified in the respective figure legends. Significance was defined as p-value/adj-p-value ≤ 0.05 if not otherwise indicated. For details see Supplementary materials and methods.

## Results

### Establishment of an in vitro MAPKi withdrawal rebound model

We tested several patient-derived in vitro models with the aim of establishing a MAPKi withdrawal rebound model fulfilling the following criteria: (1) response upon treatment (i.e. decreased cell proliferation or increased cell death); (2) cell regrowth upon treatment withdrawal; (3) cell regrowth upon MAPKi withdrawal faster than after chemotherapy withdrawal; optionally (4) faster cell proliferation after MAPKi withdrawal compared to untreated cells. Drug concentrations were chosen based on effect in vitro (Fig. S1, for details see Supplementary materials and methods section) and are within clinically relevant concentrations (Table S4).

**Fig. 1:**
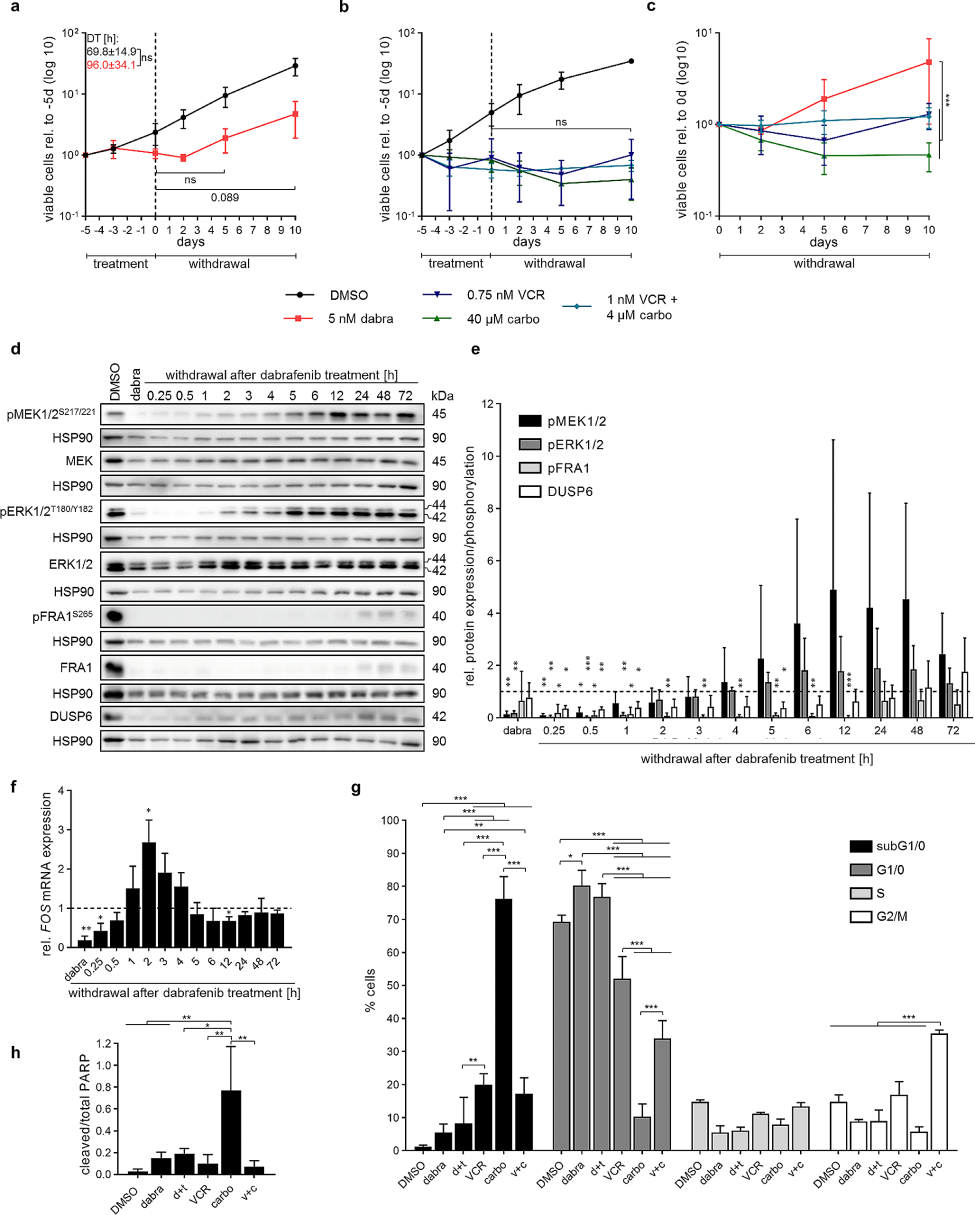
Development and characterization of an in vitro rebound model using BT-40 (*BRAF*^V600E^, *CDKN2A/B*del). (**a**-**b**) Viable cell counts during treatment and withdrawal with 5 nM dabrafenib (dabra) (**a**) or 0.75 nM vincristine (VCR), 40 µM carboplatin (carbo) and 1 nM VCR and 4µM carbo (**b**). Dashed line indicates withdrawal timepoint. Viable cell counts are normalized to treatment start (-5d). Data is shown on a logarithmic scale (base 10) as mean ± SD (*n* = 3 independent biological replicates). Doubling time (DT) was calculated from two to ten days for each biological replicate (*n* = 3) and is indicated in hours as mean ± SD. Unpaired two-sided t-test; ns: not significant. (**c**) Viable cell counts during withdrawal of cells pretreated with 5 nM dabrafenib (dabra), 0.75 nM vincristine (VCR), 40 µM carboplatin (carbo) or 1 nM VCR and 4 µM carbo for five days; viable cell counts are normalized to the withdrawal timepoint (five days of treatment) for each condition. Data is shown on a logarithmic scale (base 10) as mean ± SD (*n* = 3 independent biological). Two-way ANOVA, Bonferroni post-hoc test, *** adj-p-value ≤ 0.001; no indication: not significant. (**d**-**e**) Western blot analysis of MAPK activity markers after five days treatment with 5 nM dabrafenib (dabra) followed by treatment withdrawal. Blots shown are representative of three independent biological replicates (**d**). Quantification (**e**) is relative to solvent control (DMSO; dashed line) and shown as mean ± SD (*n* = 3 independent biological replicates). One-sample t-test, *p-value ≤ 0.05 **p-value ≤ 0.01 ***p-value ≤ 0.001; no indication: not significant. (**f**) RT-qPCR analysis of *FOS* gene expression after five days treatment with 5 nM dabrafenib (dabra) followed by treatment withdrawal. Quantification is relative to solvent control (DMSO; dashed line) and shown as mean ± SD (*n* = 3 independent biological replicates). One-sample t-test, *p-value ≤ 0.05 **p-value ≤ 0.01 ***p-value ≤ 0.001; no indication: not significant. (**g**) Cell cycle analysis using FACS after treatment for five days with 5nM dabrafenib (dabra), 2.7nM dabrafenib and 0.3nM trametinib (d + t), 0.75nM vincristine (VCR), 40µM carboplatin (carbo) or 1nM vincristine and 4µM carboplatin (v + c). Percentage of single cell population in the different cell cycle phases are shown as mean ± SD (*n* = 3 independent biological replicates). One-way ANOVA, Tukey post-hoc test, * adj-p-value ≤ 0.05 ** adj-p-value ≤ 0.01 *** adj-p-value ≤ 0.001; no indication: not significant. (**h**) Western blot quantification (of blots in Figure S2b) showing cleaved PARP relative to full-length PARP after treatment for five days with 5 nM dabrafenib (dabra), 2.7 nM dabrafenib and 0.3 nM trametinib (d + t), 0.75 nM vincristine (VCR), 40 µM carboplatin (carbo) or 1 nM vincristine and 4 µM carboplatin (v + c). Data is shown as mean ± SD (*n* = 3 independent biological replicates). One-way ANOVA, Tukey post-hoc test, *adj-p-value ≤ 0.05 **adj-p-value ≤ 0.01; no indication: not significant

Treatment of pilocytic astrocytoma (PA)-derived *KIAA1549:BRAF* fusion- (DKFZ-BT66, DKFZ-BT308) or *BRAF*^V600E^-driven cells (DKFZ-BT314) in proliferating state (induced by SV40-TAg) or senescent state (no SV40-TAg induction) did not reduce viable cell numbers compared to untreated cells (Fig. S2). Due to the lack of effect on viable cell numbers observed during treatment (i.e. rebound model criteria 1), presence or absence of rebound growth upon treatment withdrawal could not be assessed, making these models unsuitable to study the rebound growth. In contrast, treatment of the pleomorphic xanthoastrocytoma (PXA)-like cell line BT-40 (derived from a juvenile PA, without further genetic modification) [19], driven by *BRAF*^V600E^ and co-occurring *CDKN2A/B* deletion, with 5 nM dabrafenib induced a decrease of cell proliferation (Fig. 1a). Upon treatment withdrawal, cells started to re-proliferate after two days, at a similar rate as control cells (DT control:69.8 ± 14.9 h vs. DT after: 96.0 ± 34.1 h), with increased cell numbers at 10 days after withdrawal, hence fulfilling rebound model criteria 2 but not the optional criteria 4. Similar patterns were observed upon dabrafenib and trametinib combination treatment and withdrawal (Fig. S3a). Cells treated with chemotherapy (vincristine (VCR) and carboplatin), showed no cell regrowth upon withdrawal in the timeframe investigated (Fig. 1b). Accordingly, cell regrowth upon dabrafenib treatment withdrawal was significantly increased compared to SOC chemotherapy treatment (Fig. 1c, i.e. rebound model criteria 3), thereby mimicking the effect that is observed in patients and making BT-40 a suitable model to study the rebound growth.

### Characterization of the BT-40 in vitro rebound model

Molecular analysis of MAPK pathway activity in BT-40 showed near complete inhibition of the pathway after five days treatment with 5 nM dabrafenib (Fig. 1d-f). Cell cycle analysis (Fig. 1g) indicated arrest in G1/0-phase upon MAPKi treatment, without significant increase of PARP cleavage (Fig. 1h, Fig. S3b). Upon dabrafenib withdrawal, MEK and ERK phosphorylation returned to control levels after five hours (Fig. 1d-e), indicating a rapid reactivation of the pathway. MEK phosphorylation further transiently increased above control levels (Fig. 1d-e). Furthermore, DUSP6, downstream of ERK activation [20], was re-expressed at later withdrawal timepoints (24–72 h). *FOS* gene expression, an immediate target gene of ERK [21], showed a 2.5-fold increase compared to baseline two hours after withdrawal (Fig. 1f). While reactivation occurred slightly later (6 h) after combined dabrafenib and trametinib withdrawal, overall MAPK reactivation patterns were comparable (Fig. S3c-e). Importantly, DMSO withdrawal did not increase MAPK pathway activity (Fig. S3f-h), indicating the effect observed is not an artefact of the withdrawal procedure.

In contrast, as expected, SOC chemotherapy treatment and withdrawal did not induce consistent changes in the MAPK pathway, except for fluctuations in *FOS* gene expression observed after VCR withdrawal (Fig. S3i-n). Cell cycle analysis (Fig. 1g) showed an increase in the sub-G1 population in all chemotherapy treatments, indicating cell death. VCR and carboplatin combination additionally led to an increased G2/M population. Accordingly, PARP cleavage, was observed with all SOC drugs but only showed a significant increase upon carboplatin treatment (Fig. 1h, Fig. S2b), in line with the strongest induction of subG0/1 (Fig. 1g).

Taken together, these data indicate that MAPKi treatment induces cell cycle arrest with no significant cytotoxic effects, followed by a rapid reactivation of the pathway specifically upon MAPKi withdrawal.

### Multi-omics analysis reveals dynamic changes in MAPK pathway activation, cytokine expression and AKT signaling upon dabrafenib treatment and withdrawal

To investigate the underlying molecular mechanisms of regrowth after MAPKi withdrawal, we generated a multi-omics dataset from our BT-40 rebound model upon dabrafenib treatment and withdrawal, using RNA sequencing, LC-MS/MS based proteomics and phosphoproteomics.

**Fig. 2:**
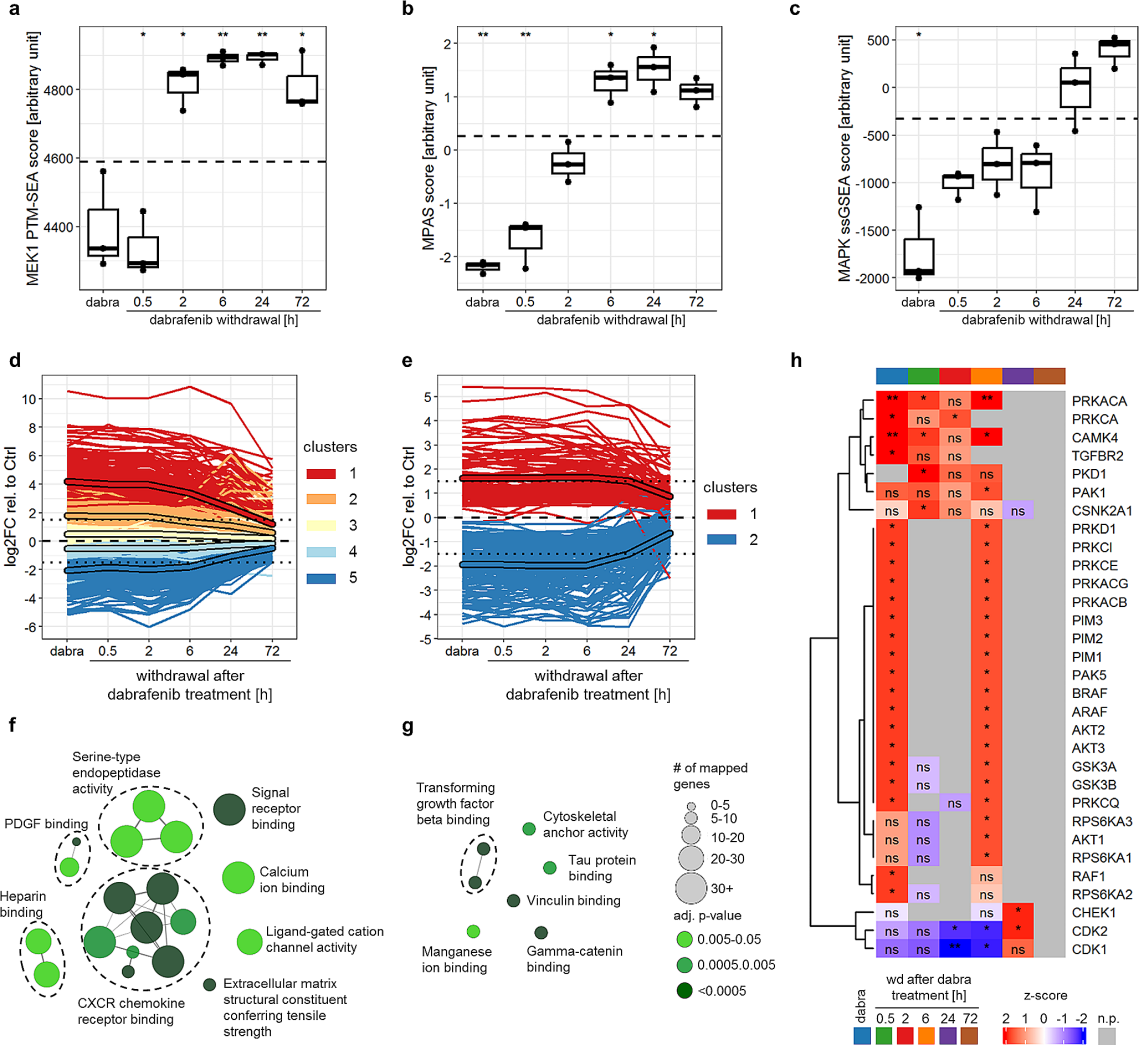
Multi-omics analysis reveals dynamic changes in MAPK pathway activation, cytokine expression and AKT signaling upon dabrafenib treatment and withdrawal. (**a**-**c**) MAPK pathway activity scores after five days treatment with 5 nM dabrafenib followed by withdrawal measured using the MEK1 PTM-SEA score [54] for phosphoproteomics data (**a**), the MPAS score [55] for RNAseq data (**b**) and a proteomics-based MAPK ssGSEA-score [56] for proteomics data (**c**). Boxplots depict the median, first and third quartiles. Whiskers extend from the hinge to the largest/smallest value no further than 1.5 *IQR from the hinge (where IQR is the interquartile range). Dashed line indicates the mean of the solvent control (five days DMSO). Two-tailed unpaired t-test, *p-value ≤ 0.05 **p-value ≤ 0.01 no indication: not significant (*n* = 3 independent biological replicates). (**d**-**e**) Longitudinal k-means clustering of differentially expressed genes (**a**) and proteins (**e**) after five days treatment with 5 nM dabrafenib followed by withdrawal relative to solvent control (five days DMSO; dashed line). Only genes and proteins with an adjusted p-value < 0.01 for at least one timepoint were included in the analysis. Unframed lines represent single genes or proteins, framed lines show clusters mean. Dotted line: log2FC = +/-1.5 (*n* = 3 independent biological replicates). (**f**-**g**) GO-term enrichment analysis of upregulated genes (clusters 1 and 2 from panel d, cluster-mean ≥ 1.5) (**f**), and proteins (cluster 1 from panel e, cluster-mean ≥ 1.5) (**g**). Only terms with significant enrichment (adj. p-value ≤ 0.05) are shown. GO-term groups are defined by overlapping genes and named based on the GO-Term with highest percentage of mapped genes. (**h**) Kinase-substrate-enrichment-analysis (KSEA) of differentially regulated phosphopeptides relative to solvent control after five days treatment with 5 nM dabrafenib (dabra) followed by withdrawal (wd). Only phospho-peptides with a significant regulation (adj. p-value < 0.01 at a given timepoint) were included in the analysis. *adj-p-value ≤ 0.05 **adj-p-value ≤ 0.01; ns: not significant; n.p.= no prediction (*n* = 3 independent biological replicates)

Decreased MAPK activity, measured using omics-specific signatures, was observed upon dabrafenib treatment (Fig. 2a-c). Upon treatment withdrawal, MAPK reactivation was observed within hours to one day (protein phosphorylation: 2 h, gene expression: 6 h, protein expression: 24 h) (Fig. 2a-c). Additionally, overactivation of the pathway during dabrafenib withdrawal was observed on the level of gene expression and protein phosphorylation (Fig. 2a-b), in line with our aforementioned data.

Longitudinal k-means clustering (kml) of differentially regulated genes, proteins and phospho-peptides showed that the majority of differential gene and protein expression or phosphorylation was induced during dabrafenib treatment, and maintained at least until 24 h of withdrawal (mean of highest differentially regulated clusters ≥ 1.5 or ≤ -1.5; Fig. 2d-e, Fig. S4a) before reaching baseline levels (= untreated control) again. Within the upregulated genes (kml-clusters 1 and 2, cluster-mean ≥ 1.5) and proteins (kml-cluster 1, cluster-mean ≥ 1.5), a significant enrichment of GO-terms related to cytokine signaling and TGF-beta signaling, respectively, was observed (Fig. 2f-g, Table S8, Table S9). Furthermore, KSEA analysis of significantly regulated phospho-peptides (adj. p-value < 0.01 at a given time-point) suggested increased activity of several signaling pathways (TGF-beta, AKT, JAK/STAT indicated by downstream effectors PIM and PAK) upon dabrafenib treatment and early withdrawal (up to 6 h; Fig. 2h).

On the other hand, decreased cell cycle activity was indicated by GO-term analysis of downregulated genes (kml-clusters 4 and 5, cluster mean ≤ -1.5) and proteins (kml-cluster 2, cluster mean ≤ -1.5) (Fig. S4b-c, Table S10, Table S11) as well as by KSEA showing decreased CDK activity (Fig. 2h). This is in line with cell cycle arrest upon treatment (Fig. 1g) and decreased cell proliferation seen until two days of withdrawal (Fig. 1a).

Taken together, these data indicate that MAPKi treatment induced the upregulation of cytokines and activates several signaling pathways (TGF-beta, AKT and JAK/STAT), potentially participating in tumor rebound upon withdrawal. Treatment withdrawal on the other hand led to the reactivation of the MAPK pathway and cell cycle related pathways, while still maintaining some of the MAPKi treatment induced changes.

### Cytokine expression and increased AKT activity are independent mechanisms without a tumor cell intrinsic effect on rebound growth

**Fig. 3:**
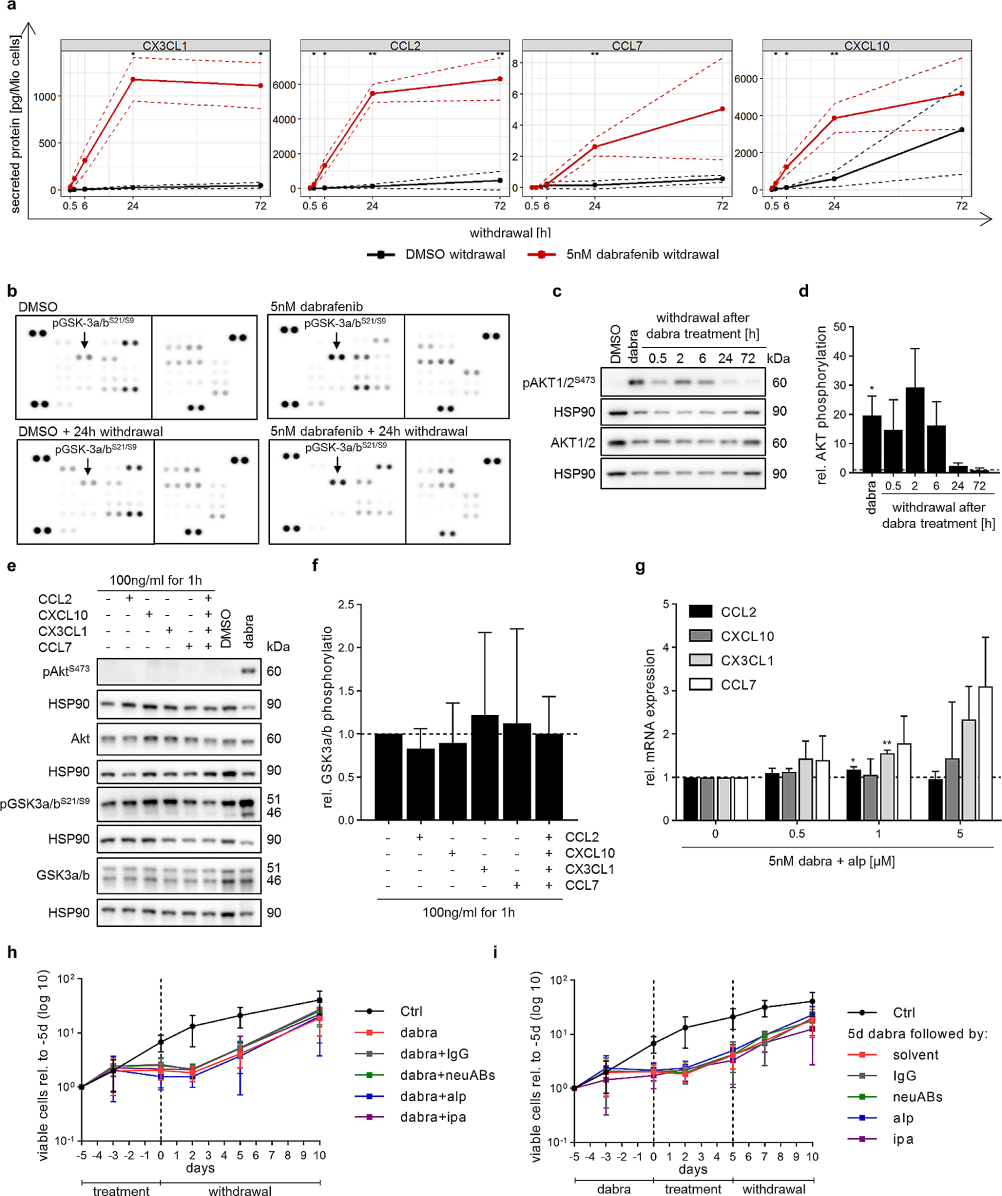
Cytokine expression and increased AKT activity are independent mechanisms without a tumor cell intrinsic effect on rebound growth. (**a**) Luminex-based multiplex assay results showing cytokine secretion during withdrawal after treatment for five days with either DMSO (solvent control) or 5 nM dabrafenib. Data is shown as mean ± SD (*n* = 3 independent biological replicates). Two-tailed unpaired t-test, *p-value ≤ 0.05 **p-value ≤ 0.01; no indication: not significant. (**b**) Kinase phosphorylation array of samples treated for five days with 5 nM dabrafenib or DMSO (solvent control) followed by 24 h withdrawal (wd). Arrays consist of two membranes, each target is detected in technical duplicates. Images shown are representatives of two biological replicates. (**c**-**d**) Western blot analysis of AKT phosphorylation after five days treatment with 5 nM dabrafenib (dabra) followed by up to 72 h withdrawal. Blots shown are representative of three independent biological replicates (**c**). Quantification (**d**) is relative to solvent control (DMSO; dashed line) and shown as mean ± SD (*n* = 3 independent biological replicates). One-sample t-test, * p-value ≤ 0.05; no indication: not significant. (**e**-**f**) Western blot analysis of AKT activity markers after treatment with recombinant cytokines. Treatment for five days with DMSO or 5 nM dabrafenib (dabra) served as negative and positive control for western blots (**e**). Blots shown are representative of three biological replicates (**e**). Quantification (**f**) was done relative to untreated samples (dashed line) and is shown as mean ± SD (*n* = 3 independent biological replicates). One-sample t-test; no indication: not significant. (**g**) RT-qPCR analysis of chemokine gene expression after five days treatment with 5 nM dabrafenib (dabra) alone or in combination with varying concentrations of alpelisib (alp). Quantification was done relative to dabrafenib only treatment (0; dashed line) and is shown as mean ± SD (*n* = 3 independent biological replicates). One-sample t-test, *p-value ≤ 0.05 **p-value ≤ 0.01; no indication: not significant. (**h**) Viable cell counts during treatment with 5nM dabrafenib (dabra) alone or in combination with a combination of antibodies neutralizing CCL2 (0.5 µg/mL), CX3CL1 (0.25 µg/mL), CXCL10 (0.25 µg/mL) and CCL7 (0.1 ng/mL) (neuABs), IgG control (1 µg/mL), 5 µM alpelisib (alp) or 1 µM ipatasertib (ipa) followed by ten days of withdrawal. Dashed line indicates withdrawal timepoint. Viable cell counts are normalized to treatment start (-5d). Data is shown on a logarithmic scale (base 10) as mean ± SD of at least three biological replicates. (**i**) Viable cell counts during treatment with 5 nM dabrafenib (dabra) followed by withdrawal. During withdrawal cells were either untreated (= solvent) or treated for five days with a combination of antibodies neutralizing CCL2 (0.5 µg/mL), CX3CL1 (0.25 µg/mL), CXCL10 (0.25 µg/mL) and CCL7 (0.1 ng/mL) (neuABs), IgG control (1 µg/mL), 5 µM alpelisib (alp) or 1 µM ipatasertib (ipa), followed by five days of withdrawal. Dashed lines indicate withdrawal timepoints. Viable cell counts are normalized to treatment start (-5d). Data is shown on a logarithmic scale (base 10) as mean ± SD of at least three biological replicates

We next investigated the secretion of cytokines showing increased gene expression (21 cytokines; log2FC > 2, padj < 0.01 at 6 h of withdrawal; Fig. S4d) using a commercially available Luminex-based multiplex assay (covering 16/21 cytokines). Cytokine secretion was increased upon dabrafenib treatment and maintained upon withdrawal (Fig. S5a), with 11/16 cytokines maintaining increased secretion with a > 2-fold increase compared to DMSO withdrawal (AUC-log2FC > 1; Table S12). Of these eleven, secretion of CCL2, CX3CL1, CCL7 and CXCL10 was significantly higher 24 h after dabrafenib withdrawal compared to DMSO, one day before cells are proliferating again (Fig. 3a, Fig. S5b).

To validate the differential regulation of the aforementioned pathways, a phosphorylation kinase array and western blot analysis was used. Increased AKT activity (GSK3-a/b and AKT phosphorylation) upon treatment and withdrawal was confirmed (Fig. 3b-d, Fig. S6c), but no increased TGF-beta or JAK/STAT signaling was observed (Fig. S6a-c).

Of note, increased expression of *CCL2, CX3CL1, CCL7* and *CXCL10*, and increased AKT activity, were also observed for dabrafenib and trametinib combination treatment and withdrawal (Fig. S6d-f).

We next investigated a possible interplay between CCL2, CX3CL1, CCL7 and CXCL10 expression and AKT activation. While some cytokine receptors were expressed in BT-40 (Fig. S7a), neither stimulation with recombinant cytokines (Fig. 3e-f) nor inhibition of cytokines using neutralizing antibodies (neuABs, Fig. S7b-c) affected AKT activity. Furthermore, inhibition of AKT activity using the PI3K inhibitor alpelisib did not suppress dabrafenib-induced cytokine expression (Fig. 3g, Fig. S7d-e). Overall, this indicates independence of MAPKi-induced cytokine expression from AKT signaling in our model.

While both cytokines and AKT signaling independently can be survival- and growth-promoting, neither cytokine inhibition by neuABs nor AKT inhibition by alpelisib (PI3Ki) or ipatasertib (AKTi) during MAPKi treatment or withdrawal reduced viable cell counts compared to MAPK inhibition only (Fig. 3h-i, Fig. S7d-j).

Taken together these data indicate the absence of a tumor cell intrinsic growth-promoting effect of the cytokines or AKT signaling during MAPKi treatment or withdrawal, and suggests that MAPK pathway reactivation is the natural driver of the intrinsic tumor rebound mechanism in our rebound model.

### In vivo validation of MAPK pathway reactivation during rebound growth

**Fig. 4:**
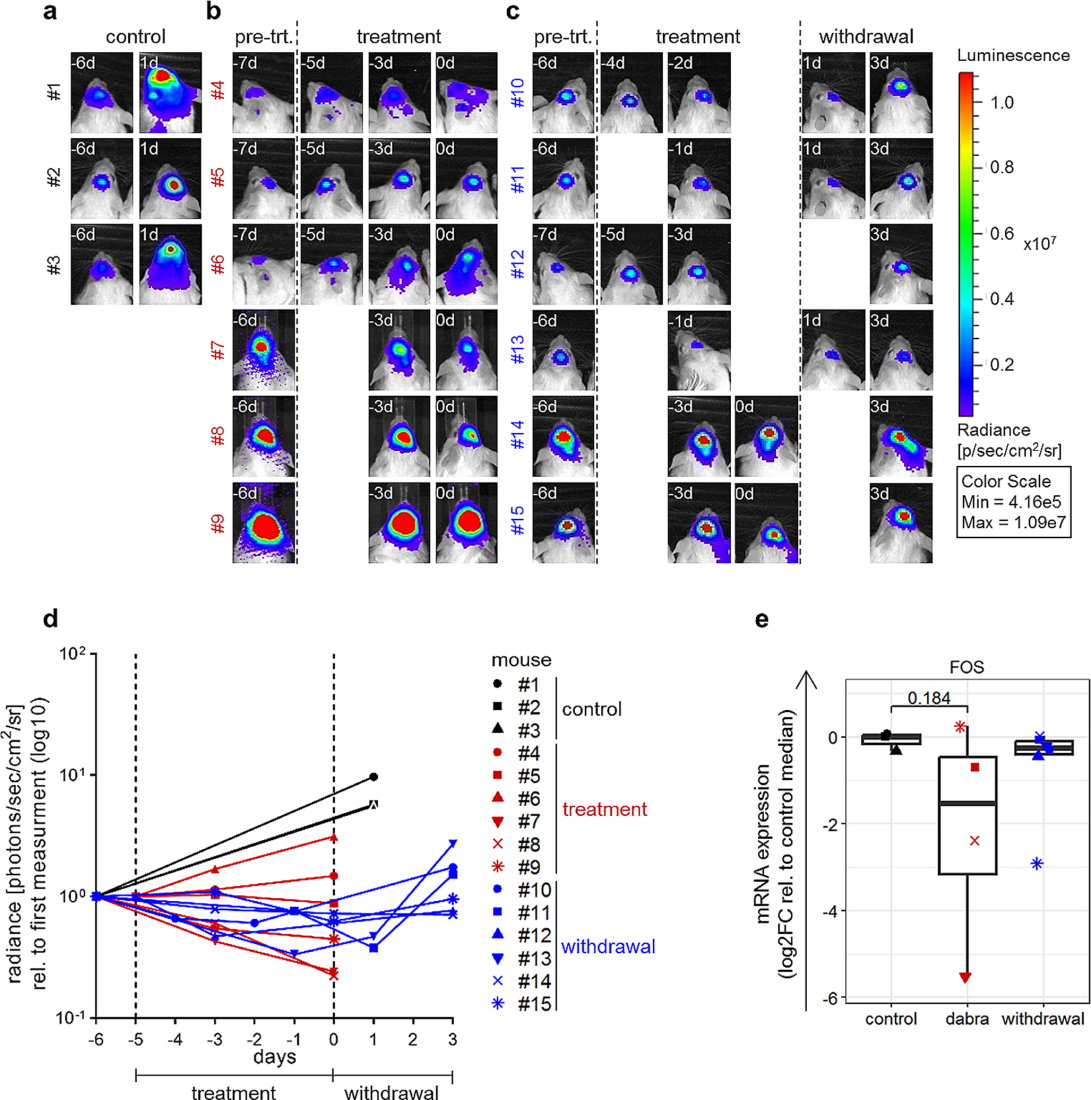
MAPK reactivation upon dabrafenib treatment and withdrawal in BT-40 orthotopic xenograft model. (**a**-**d**) NSG mice carrying BT-40 xenograft tumors were treated with 100 mg/kg dabrafenib (treatment; six doses, once daily) followed by three days of withdrawal. Tumor growth was measure by bioluminescence imaging and is shown for each individual animal as radiance in photons/s/cm²/steradian (**d**). Dashed lines indicate treatment start and treatment stop. Bioluminescence images (a-c) of untreated animals (control, *n* = 3; a), animals undergoing treatment (*n* = 6; b) and animals undergoing treatment followed by withdrawal (*n* = 6; c); pre-trt. = before treatment start. (**e**) RT-qPCR analysis of *FOS* expression in BT-40 xenograft tumors after six days treatment with dabrafenib (dabra; 100 mg/kg, six doses, once daily) followed by three days of withdrawal (withdrawal). Samples from mice showing tumor progression (#4, #6; panel d) during dabrafenib treatment were excluded from the analysis. Quantification was done relative to the median of untreated samples (control). *FOS* was undetected in two samples (#7, #8), for these samples Ct values were set to 40 (max. number of cycles). Boxplots depict the median, first and third quartiles. Whiskers extend from the hinge to the largest/smallest value no further than 1.5 *IQR from the hinge (where IQR is the interquartile range). Two-tailed unpaired t-test; no indication: not significant (control: *n* = 3 mice, dabra: *n* = 4 mice, withdrawal: *n* = 6 mice)

To investigate the MAPK pathway reactivation upon treatment withdrawal in vivo, BT-40 cells were engrafted orthotopically in NOD scid gamma (NSG) mice to generate samples for molecular analysis (gene expression) during treatment and withdrawal. As shown by bioluminescent imaging, used to control for treatment effect, 100 mg/kg dabrafenib treatment stabilized tumor growth within six days of treatment in 10/12 mice (non-responding mice were excluded from further analysis). Upon treatment stop, a trend for increased tumor size was observed during the first three days of withdrawal and before harvesting of tumors in 2/6 mice, suggesting the possibility of rebound growth in our in vivo model (Fig. 4a-d).

Gene expression analysis of tumor samples showed decreased *FOS* expression during treatment and re-expression during treatment withdrawal compared to untreated controls (Fig. 4e), in line with MAPK pathway regulation patterns observed in vitro.

### MAPKi-induced cytokines attract microglia in a paracrine fashion

**Fig. 5:**
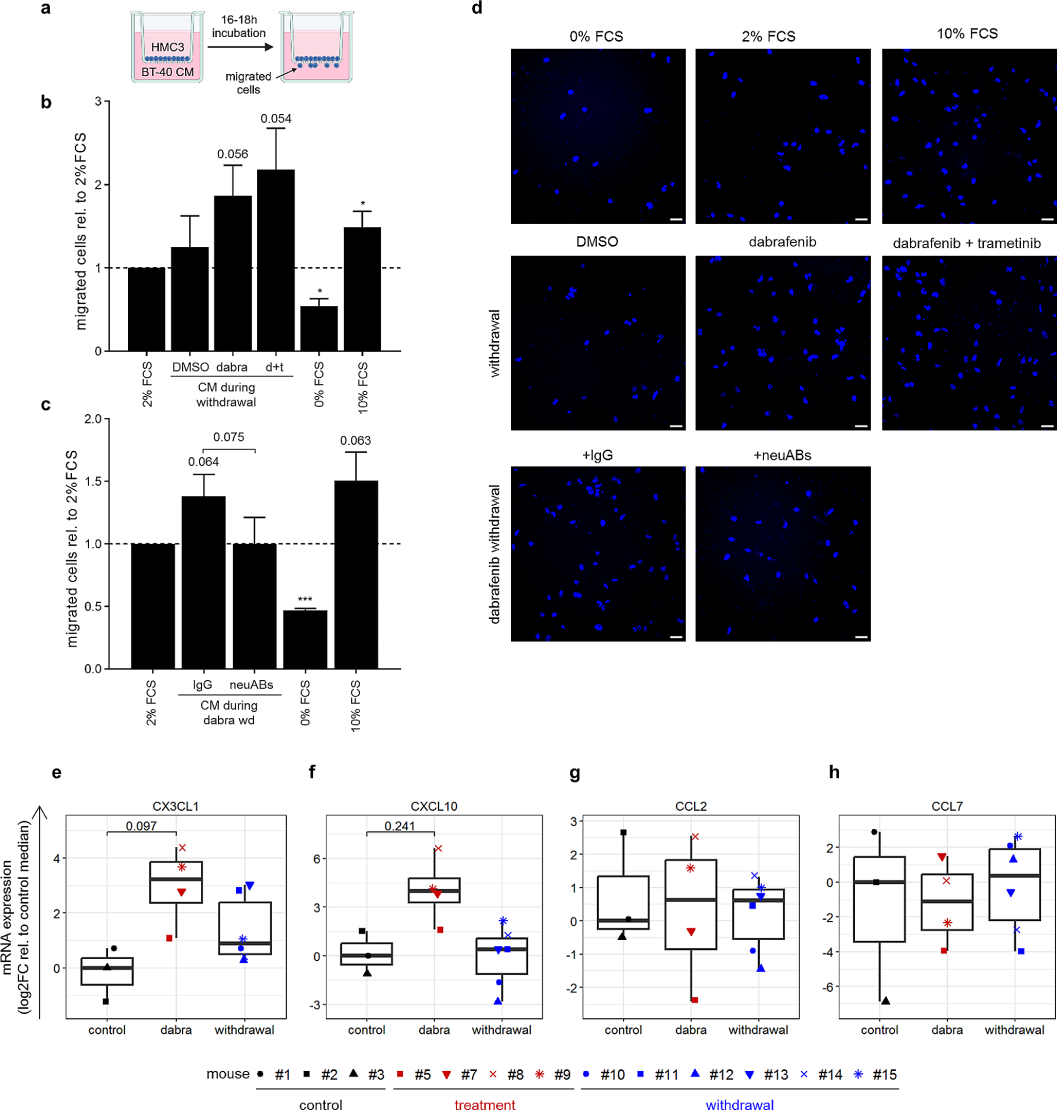
Increased cytokine secretion upon MAPKi treatment and withdrawal induces increased microglia attraction. (**a**) Schematic of transwell migration assay setup to investigate migration of HMC3 cells towards conditioned media (CM) collected from BT-40. (**b**) Transwell migration assay of HMC3 cells towards conditioned media (CM) collected from BT-40 cells after 24 h of withdrawal after five days treatment with DMSO (solvent control), 5nM dabrafenib (d) or 2.7 nM dabrafenib and 0.3 nM trametinib (d + t). 2% FCS serves as baseline control as CM contains 2% FCS, 0% FCS as negative control and 10% FCS as positive control. Quantification is shown as mean ± SD (*n* = 3 independent biological replicates; 2 technical duplicates per condition; 10–12 randomly distributed images were quantified per transwell) relative to 2% FCS. One-sample t-test, * p-value ≤ 0.05; no indication: not significant. (**c**) Transwell migration assay of HMC3 cells towards CM collected 24 h after dabrafenib withdrawal (dabra wd) containing either antibodies neutralizing CCL2 (1 µg/mL), CX3CL1 (0.5 µg/mL), CXCL10 (0.5 µg/mL) and CCL7 (0.2 ng/mL) (neuABs) or IgG (2 µg/mL). 2% FCS serves as baseline control as CM contains 2% FCS, 0% FCS as negative control and 10% FCS as positive control. Quantification is shown as mean ± SD (*n* = 3 independent biological replicates; 2 technical duplicates per condition; 10–12 randomly distributed images were quantified per transwell) relative to 2% FCS. One-sample t-test and two-tailed unpaired t-test (comparing IgG to neuABs), *p-value ≤ 0.05 ***p-value ≤ 0.001; no indication: not significant. (**d**) Representative fluorescence images showing HMC3 migrated through the transwell, nuclei were stained with DAPI. Scale bar = 50 µM. (**e**-**h**) RT-qPCR analysis of *CX3CL1* (**e**), *CXCL10* (**f**), *CCL2* (**g**) and *CCL7* (**h**) expression in BT-40 xenograft tumors after six days treatment with dabrafenib (dabra; 100 mg/kg, six doses, once daily) followed by three days of withdrawal (withdrawal). Samples from mice showing tumor progression (#4, #6; Fig. 4d) during dabrafenib treatment were excluded from the analysis. Quantification was done relative to the median of untreated samples (control). *CX3CL1* was undetected in one sample (#2), *CCL2* was undetected in two samples (#2, #7) and *CCL7* was undetected in six samples (#2, #3, #5, #7, #8, #11), for these samples Ct values were set to 40 (max. number of cycles). Boxplots depict the median, first and third quartiles. Whiskers extend from the hinge to the largest/smallest value no further than 1.5 * IQR from the hinge (where IQR is the interquartile range). Two-tailed unpaired t-test; no indication: not significant (control: *n* = 3 mice, dabra: *n* = 4 mice, withdrawal: *n* = 6 mice)

Since the cytokines had no tumor cell intrinsic effects, we hypothesized that they might have tumor extrinsic properties, that could further increase tumor growth (i.e. optional rebound model criteria 4), which was not observed in BT-40 monoculture. As cytokines, in particular CCL2 and CX3CL1, have been described to attract microglia cells [22–24], we investigated microglia attraction using conditioned media (CM; Fig. 5a). Indeed, increased migration of HMC3 cells, with a trend towards statistical significance, was observed using CM collected from BT-40 cells during MAPKi withdrawal (Fig. 5b, d), which was reversed by the addition of a combination of antibodies neutralizing CCL2, CX3CL1, CXCL10 and CCL7 (Fig. 5c-d). Additionally, we could show that microglia cells support BT-40 growth (Fig. S8), suggesting that microglia cells recruited upon treatment withdrawal could increase tumor growth (i.e. optional tumor rebound criteria 4). Importantly, a trend for increased mRNA expression of *CX3CL1* and *CXCL10* in tumor cells was also observed upon dabrafenib treatment in vivo (Fig. 5e-h), suggesting that such a recruitment could take place in vivo. Further investigation in pLGG PDX-models in immunocompetent mice, yet to be established [25], will be necessary to validate these findings.

Of note, increased microglia attraction was also observed using conditioned media collected during MAPKi treatment (Fig. S9). The role of microglia in the treatment period remains to be elucidated.

Taken together, these data suggest a possible tumor cell extrinsic, paracrine mechanism of MAPKi-induced cytokine secretion involving microglia cells, warranting further investigation using more complex models.

## Discussion

Rebound growth of pLGGs after MAPKi treatment remains a clinical challenge and is not well understood. Both prediction as well as prevention of rebound growth need a deeper molecular and biological understanding of the underlying mechanisms driving it.

We successfully modeled the rebound growth of pediatric glioma upon MAPKi withdrawal in vitro using BT-40 (*BRAF*^V600E^, *CDKN2A/B*del). However, in additional pLGG models driven by *KIAA1549:BRAF* fusion (DKFZ-BT66, DKFZ-BT308) or *BRAF*^V600E^ mutation (DKFZ-BT314) we did not observe an effect on viable cell counts during treatment, in line with previous data [14, 15, 26]. This may be explained by inhibition of p53 and pRB through expression of SV40-TAg [14, 27, 28] in the proliferating cells and by the biology of senescent cells, which commonly do not undergo apoptosis [15, 26, 29]. Due to the lack of effect during MAPKi treatment in these models, the presence or absence of rebound growth could not be assessed and these models could not be further used in this study. Nonetheless, our BT-40 rebound model represents an important pediatric glioma subgroup, as patients with this genetic background have the poorest prognosis [30]. Whether our findings in BT-40 also apply to pLGG entities with different genetic backgrounds needs to be further validated in future studies using more suitable models, including non-rebounding models as additional control.

Using our BT-40 rebound model, we observed rapid reactivation of the MAPK pathway upon MAPKi withdrawal. As rebound growth in pLGG is not associated with MAPKi resistance [8, 13], a MAPK-dependent driving mechanism is plausible and we assume that the fast MAPK reactivation plays a role in the rebound growth. In addition to a rapid reactivation, we observed a transient overshooting activation of the pathway, which has also been observed in other MAPK-driven tumor entities [31–33]. Interestingly, in our model, MAPK overactivation was most prominently observed on the level of MEK phosphorylation and MAPK target gene expression (*FOS*, MPAS score). Substantial pERK rebound, as observed in other studies and associated with reduced proliferation and cell death in MAPKi addiction models [31–33], was not observed, likely due to the parallel re-expression of DUSP6, which negatively regulates ERK activity [34]. Taken together, this indicates a potential vulnerability to DUSP6 inhibitors, as targeting of this phosphatase may induce apoptosis due to overshooting MAPK activity [35, 36].

In addition to a fast reactivation of the MAPK pathway, we observed upregulation of AKT signaling, which is commonly observed upon MAPKi in other entities [37–39] and combination of MAPKi and PI3K/AKT/mTORi was shown to have beneficial effects in different tumor entities [40–42], including pLGGs [43]. In contrast to what was previously described for BT-40 [43], inhibition of AKT signaling in combination with MAPKi did not further decrease viable cell counts compared to MAPKi only, and most importantly it did not affect rebound growth in our setting. This could be explained by the fact that the synergistic activity of mTORCi and MEKi involved effects mediated by the microenvironment (i.e. angiogenesis) [43], which is not reflected in our in vitro setting.

Our analysis furthermore revealed increased expression and secretion of several cytokines, in particular CCL2, CX3CL1, CXCL10 and CCL7, upon dabrafenib treatment and withdrawal in vitro, with a trend for increased expression of *CX3CL1* and *CXCL10* also in vivo. While cytokines have been described to have direct growth-promoting effects on tumor cells [44–47], in our rebound model, inhibition of these cytokines had no effect on BT-40 viability or proliferation, indicating that these cytokines have no tumor cell intrinsic effect. However, we were able to demonstrate a tendency for increased attraction of microglia cells, which make up the majority of the TME in pLGG tumors [3]. In line with previous work demonstrating the role of cytokines in immune cell attraction [22–24, 48–51], a trend for decreased microglia attraction was observed upon inhibition of CCL2, CX3CL1, CXCL10 and CCL7 in our setting.

Increased microglia attraction by the tumor cells upon MAPKi withdrawal observed in vitro could possibly contribute to a neuroradiologically growing tumor mass, as observed on MRI. Furthermore, we observed growth-promoting effects of microglia on BT-40 cells in vitro. In line, a study using *BRAF* fusion-driven neural stem cells showed the importance of microglia recruitment for tumor formation in vivo [52]. This suggests a potential tumor growth-promoting role of microglia cells (attracted either during MAPKi treatment and/or immediately after withdrawal while cytokines are still increased), possibly enhancing tumor growth after MAPKi withdrawal and leading to faster growth after compared to before treatment. Furthermore, attraction of microglia cells may have implications during MAPKi treatment, as microglia infiltration in pLGG tumors correlated with a high predictive MAPK inhibitor sensitivity score (MSS) [53]. Taken together, these data indicate a possible complex role of microglia in response to MAPKi treatment and rebound growth after treatment withdrawal in pLGG. The exact role of microglia cells and how their activation status (i.e. tumor-promoting or -suppressive) may be influenced by MAPKi treatment and withdrawal needs further investigation, for which more complex models, such as organoids containing immune cells or immunocompetent mouse models [25], will be necessary.

In summary, we have identified the MAPK pathway as a tumor cell intrinsic rebound driving mechanism in pediatric glioma cells with *BRAF*^V600E^ mutation and *CDKN2A/B*del. In addition, MAPKi withdrawal was associated with the expression and secretion of microglia-recruiting cytokines, which may contribute to rebound growth in a paracrine manner. Further validation of our findings in patient samples will be necessary. Such samples remain to date extremely scarce, since re-challenging with MAPKi is currently favored to surgery to treat rebounding patients (clinical communication). Nonetheless, our data opens the question whether further development of different MAPK inhibitors for the treatment of pediatric glioma is enough to sufficiently improve patient outcome, as the MAPK pathway will most likely be reactivated with any inhibitor upon treatment stop. Therefore, the exploration of more effective treatment strategies, possibly involving modulation of the tumor immune microenvironment, suggested to play a role by our data, are warranted to improve patient’s quality of life.

### Electronic supplementary material

Below is the link to the electronic supplementary material.

Supplementary Material 1

Supplementary Material 2

Supplementary Material 3

## Data Availability

The RNA sequencing data (GSE249718) generated in this study is available from the GEO platform. The LC-MS/MS proteomics and phosphoproteomics data (PXD048277) generated in this study are available from the PRIDE database.
