# Supplementary material for: Rebound growth of BRAF mutant pediatric glioma cells after MAPKi withdrawal is associated with MAPK reactivation and secretion of microglia-recruiting cytokines
Source: J Neurooncol. 2024 Apr 17;168(2):317–32. doi: 10.1007/s11060-024-04672-9 (PMC11147834; doi:10.1007/s11060-024-04672-9)
Supplement: Supplementary file 1 — Supplementary Material 1 [file 11060_2024_4672_MOESM1_ESM.pdf]

Rebound growth of BRAF mutant pediatric glioma cells after MAPKi withdrawal is associated with MAPK reactivation and secretion of microglia-recruiting cytokines

Daniela Kocher[1,2,3,4], Lei Cao[5,6], Romain Guiho[5,7], Melanie Langhammer[8,9], Yun-Lu Lai[1,2,3], Pauline Becker[1,2,3,10], Hiba Hamdi[5], Dennis Friedel[4,11,12], Florian Selt[1,2,3,13], David Vonhören[4,11,12], Julia Zaman[4,11,12], Gintvile Valinciute[1,2,3], Sonja Herter[1,2,3,4], Daniel Picard[14,15,16], Johanna Rettenmeier[1,2,10,17,18], Kendra K. Maass[1,2,17,18], Kristian W. Pajtler[1,2,13,17,18], Marc Remke[19], Andreas von Deimling[11,12], Stefan Pusch[11,12], Stefan M. Pfister[1,2,13,17], Ina Oehme[1,2,3], David T.W. Jones[1,2,20], Sebastian Halbach[8,21], Tilman Brummer[8,21,22], Juan Pedro Martinez-Barbera[5], Olaf Witt[1,2,3,13], Till Milde[1,2,3,13,**], Romain Sigaud[1,2,3,*]

[1]Hopp Children's Cancer Center Heidelberg (KiTZ), Heidelberg, Germany
[2]National Center for Tumor Diseases (NCT), NCT Heidelberg, a partnership between DKFZ and Heidelberg University Hospital, Germany.
[3]German Cancer Research Center (DKFZ) Heidelberg, Clinical Cooperation Unit Pediatric Oncology, Germany
[4]Faculty of Biosciences, Heidelberg University, Heidelberg, Germany
[5]Developmental Biology and Cancer Programme, Birth Defects Research Centre, Great Ormond Street Institute of Child Health, University College London, 30 Guilford Street, London, WC1N 1EH, UK
[6]Department of Neurosurgery, Beijing Tiantan Hospital, Capital Medical University, Beijing, China
[7]Nantes Université, Oniris, INSERM, Regenerative Medicine and Skeleton, RMeS, UMR 1229, F-44000 Nantes, France
[8]Institute of Molecular Medicine and Cell Research (IMMZ), Faculty of Medicine, University of Freiburg
[9]Faculty of Biology, University of Freiburg, Freiburg, Germany
[10]Faculty of Medicine, Heidelberg University, Heidelberg, Germany.
[11]Department of Neuropathology, Heidelberg University Hospital, Heidelberg, Germany
[12]German Cancer Research Center (DKFZ), Clinical Cooperation Unit Neuropathology , Heidelberg, Germany
[13]KiTZ Clinical Trial Unit (ZIPO), Department of Pediatric Hematology and Oncology, Heidelberg University Hospital, Heidelberg, Germany
[14]Department of Pediatric Oncology, Hematology, and Clinical Immunology, Medical Faculty, and University Hospital Düsseldorf, Heinrich Heine University, Düsseldorf, Germany
[15]German Cancer Consortium (DKTK), Partner site Essen/Düsseldorf, Düsseldorf, Germany
[16]Institute of Neuropathology, Medical Faculty, and University Hospital Düsseldorf, Heinrich Heine University, Düsseldorf, Germany
[17]German Cancer Research Center (DKFZ), Division of Pediatric Neurooncology, Heidelberg, Germany
[18]Department of Pediatric Oncology, Hematology, Immunology and Pulmonology, Heidelberg University Hospital, Heidelberg, Germany
[19]Pediatric Hematology and Oncology, University Children's Hospital, Saarland University, Homburg, Germany
[20]German Cancer Research Center (DKFZ) Heidelberg, Division of Pediatric Glioma Research, Germany
[21]German Consortium for Translational Cancer Research (DKTK), Freiburg, Germany, German Cancer Research Center (DKFZ), Heidelberg, Germany
[22]Centre for Biological Signaling Studies BIOSS, University of Freiburg

*Corresponding author: Dr. Romain Sigaud, Hopp Children's Cancer Center Heidelberg (KiTZ), Im Neuenheimer Feld 280, 69120, Heidelberg, Germany (r.sigaud@kitz-heidelberg.de)

**Corresponding author: Prof. Dr. med. Till Milde, Hopp Children's Cancer Center Heidelberg (KiTZ), Im Neuenheimer Feld 280, 69120, Heidelberg, Germany (t.milde@kitz-heidelberg.de)

SUPPLEMENTARY MATERIALS & METHODS

Cell culture

For BT-40 cells, MM was RPMI with 2% FCS. For, DKFZ-BT308 and DKFZ-BT314, MM was ABM (cat. no. CC-3187, Lonza) with only 2% FCS and without the EGF and insulin supplements (cat.no. CC-3186, Lonza). For DKFZ-BT66, experiments were performed in complete medium.

All cells were cultured in a humidified incubator at 37 °C and 5% CO2, dissociated using 0.05% Trypsin-EDTA (cat. no. 25300054, ThermoFisher Scientific) and counted with a Vi-CELL XR (Beckman Coulter; Software v2.03) using the settings described in Table S1.
Seeding densities for each cell line for different experiments are listed in Table S2.

Cell lines were authenticated through SNP or STR profiling using Multiplex Cell Authentication by Multiplexion GmbH (Heidelberg, Germany). The purity of cell lines was evaluated using the Multiplex cell Contamination Test by Multiplexion GmbH (Heidelberg, Germany).

Drug treatments and withdrawal

Drug concentrations for MAPKi were chosen based on effect *in vitro* as measured by a metabolic activity assay in BT-40 (Fig. S1) or a MAPK reporter assay [1] in DKFZ-BT66 [2].
For any drug, concentrations chosen do not exceed maximum plasma concentrations (Cmax) [3–6]. If possible, plasma-protein-binding (PPB) [7] was taken into account and unbound Cmax concentrations ($unbound\ Cmax = Cmax\ \times \frac{100-PPB}{100}$) were used (Table S4).

For treatment withdrawal, cells were washed three times with PBS (cat. no. D8537, Sigma-Aldrich), incubated for 15 min in medium at 37 °C, followed by three PBS washes and lastly addition of fresh medium. Time of withdrawal is counted from the timepoint fresh medium is added after the last PBS wash.

Metabolic activity assay for IC50 determination

For metabolic activity assays, BT-40 were seeded in RPMI containing 2% FCS. One day after seeding, cells were treated using the D300e Digital Dispenser (Tecan). 72 h after treatment start, metabolic activity was measured using CellTiter-Glo 2.0 (cat. no. G9241, Promega) according to the manufacturer's instructions. Luminescence signal was measured using the FLUOstar OPTIMA automated plate reader (BMG Labtech). IC50/IC75 values were calculated using GraphPad Prism (v8.0.2) with a 4-parameter dose-response model.

Cell counting for growth curve analysis

The mean viable cell number of two technical replicates for each time point and condition was calculated and plotted. Doubling time (DT) was calculated as follows:

$DT = \frac{t \times log10(2)}{log10(X_{end}) - log10(X_{start})}$ where t refers to the time between the first cell count and the last cell count and X_{start} and X_{end} refer to the viable cell numbers at the first and last cell count respectively. For the DT calculation of untreated cells, time-frames for calculation where chose to lie within the exponential growth phase of the cells (i.e. before plateau of the growth curve due to contact inhibition)

RNA isolation, cDNA synthesis and quantitative reverse transcription real-time PCR (RT-qPCR)

RNA extraction was performed using the RNeasy Mini Kit (cat. no. 74104, Qiagen) with on-column DNase digestion according to the manufacturer's instructions. In case of tumor tissue samples, the TissueLyser II (Qiagen) was used for mechanical tissue dissociation and lysis according to the manufacturer's instructions. cDNA synthesis was done using the RevertAid First Strand cDNA Synthesis Kit (cat. no. K1622, ThermoFisher Scientific) following the manufacturer's protocol. qPCR was performed as described previously [8] using an ABI 7500 Real Time PCR cycler (Applied Biosystems) with ABI 7500 Software v2.3 (Applied Biosystems) and qPCR Mastermix for

SYBR® Green I (cat. no. 4309155, ThermoFisher Scientific). The ΔΔCt method was used for relative quantification. *ACTB* and *TBP* were used as housekeeping genes for all *in vitro* samples. For *in vivo* xenograft tumor samples only *ACTB* was used. In case genes of interest were undetected in some samples, Ct values for these samples were set to 40 (max. number of cycles).

Protein extraction and immunoblotting

Cells were lysed in SDS-Buffer containing PhosSTOP phosphatase inhibitors (cat. no. 49068450001, Sigma Aldrich) and cOmplete™ mini proteinase inhibitors (cat. no. 11836153001, Sigma Aldrich). Protein concentration was measured with the Pierce™ BCA Protein Assay Kit (cat. no. 23227, ThermoFisher Scientific) using the FLUOstar OPTIMA automated plate reader (BMG Labtech). Gel electrophoreses was performed using 7% or 10% acrylamide gels. Proteins were transferred to a PVDF membrane using the Trans-Blot Turbo RTA Mini 0.45 µM LF PVDF Transfer Kit (cat. no. 1704274, Biorad) with the Trans-Blot Turbo Transfer System (Biorad). Immunodetection was done with Amersham ECL Prime Western Blotting Detection Reagent (cat. no. RPN2232, GE Healthcare Dharmacon) using the Azure c400 imaging system (Azure Biosystems). Quantification was done using ImageJ (v2.9.0)

RNA sequencing and data processing

RNA sample integrity after isolation was assessed using the 2100 Bioanalyzer (Aligent). Sequencing libraries were prepared using the Illumina TruSeq mRNA stranded Kit following the manufacturer's instructions. Briefly, mRNA was purified from 500 ng of total RNA using oligo(dT) beads. Then poly(A)+ RNA was fragmented to 150 bp and converted to cDNA. The cDNA fragments were then end-repaired, adenylated on the 3′ end, adapter ligated and amplified with 15 cycles of PCR. The final libraries were validated using Qubit (Invitrogen) and TapeStation (Agilent Technologies). 2x 100 bp paired-end sequencing was performed on the Illumina NovaSeq 6000 according to the manufacturer's protocol. FASTq files were then submitted to the OTP RNAseq pipeline[9] for read-trimming, alignment to the hg19/GRCh37 human genome and

calculation of raw read counts, TPM and FPKM values. Afterwards, data was sorted to only include protein-coding genes. Furthermore, lowly expressed genes (TPM<1 in all samples) were excluded.

LC-MS/MS proteomics and phosphoproteomics data generation and processing

Cell were lysed in 4% SDC buffer, boiled at 95°C and sonicated using a tip probe sonicator (1s pulses, 40% power for 1min). Protein concentration was measured with the Pierce™ BCA Protein Assay Kit (cat. no. 23227, ThermoFisher Scientific) using the FLUOstar OPTIMA automated plate reader (BMG Labtech).

For proteomics analysis, 50 µg protein was used. 400 mM 2-Chloracetamide, 100 mM Tris-(2-carboxyethyl)-phosphin hydrogen chloride and 400 mM potassium hydroxide were used to reduce and alkylate cysteine residues. Proteins were digested using Trypsin/Lys-C mix (cat. no. V5072, Promega) overnight (16-18 h) at 37°C and 1400 rpm, followed by inactivation using 1% TFA. 20 µg of digested protein was then used for peptide clean up on self-made SDB-PRS stage tips, prepared by stuffing a 200 µL pipette tip (without filter) with 3 layers of an SDB-RPS extraction disk (Merck) using a modified blunt-end syringe (5 mL, 14 gauge). For centrifugation, a 3D-printed adapter was used. Stage tips were equilibrated prior to peptide loading using acetonitrile, followed by 30% methanol, followed by 0.2% trifluoroacetic acid in water. After peptide loading, washing was performed using 1% trifluoroacetic acid in 2-propanol followed by 0.2% trifluoroacetic acid in water. Finally, peptides were eluted using 80% acetonitrile and 1.25% ammonium hydroxide in H_2O, dried using a vacuum concentrator at 45 °C and suspended in 2.5% 1,1,1,3,3,3-Hexafluoro-2-propanol and 0.1% trifluoroacetic acid in H_2O. Peptide concentration was determined using the Pierce™ Quantitative Colorimetric Peptide Assay Kit (cat. no. 23275, ThermoFisher Scientific) after sonication in an ultrasound bath (15 min) and a total amount of 0.5 µg was subjected to LC-MS/MS analysis.

For phosphoproteomics analysis, 500 µg protein was used. 10 mM DTT and 500 mM iod-acetamide were used to reduce disulfide-bonds and alkylate cysteine residues. Samples were cleaned by acetone precipitation before protein digestion overnight (16-18 h) using Trypsin/Lys-C mix (cat. no. V5072, Promega). Trypsin was inactivated using formic acid and peptides were dried using a vacuum concentrator at 45 °C. Afterwards, phosphopeptides were enriched using the High-Select™ TiO2 Phosphopeptide Enrichment Kit (cat. no. A32993, ThermoFisher Scientific) according to the manufacturer's instructions. After enrichment, peptides were dried using a vacuum concentrator at 45 °C, resuspended in 2.5% 1,1,1,3,3,3-Hexafluoro-2-propanol and 0.1% trifluoroacetic acid in H_2O and subjected to LC-MS/MS analysis

Prior to MS/MS analysis using a timsTOF pro mass spectrometer (Bruker), samples were separated by a NanoElute HPLC system using a 90 min gradient. Ion accumulation and ramp time were set to 50 ms, ions with a mobility ranging from 1/K0 = 0.85-1.3 V s cm^{-2} were included. Precursors reaching an intensity threshold of 1500 arbitrary units (a.u.) were classified as suitable. Resequencing of low-abundance precursors was performed, taking dynamic exclusion of 40 s into account, until a value of 20000 a.u. was reached. Ions with a mass range = 100-1700 m/z were selected for MS/MS fragmentation. A 2 Th window or a 3 Th window was used for ions with m/z < 700 or mz > 700 respectively. Quadropole switching events were synchronized with the precursor elution profile for isolation. Collision energy for dissociation was lowered linear as a function of increasing ion mobility, ranging from 59 eV (1/K0 = 1.6 V s cm^{-2}) to 20 eV (1/K0 = 0.6 V s cm^{-2}). Single charged precursor ions were excluded using a polygon filter.

Raw MS data was processed by the commandline version of the MaxQuant software (v1.6.17.0) [10] on a Linux machine with 128 physical cores (AMD EPIC 75032 32-Core Processor) and 256Gb of RAM. Spectra were searched against the human Uniprot database of canonical protein sequences downloaded in March 2022. Parameters including enzyme specificity, FDR on peptide spectral match, protein level precursor as well as fragment ion mass tolerance remained on default settings. For whole proteome analysis the variable modifications Aminoacid deamidiation (NQ),

Oxidation (M) and Acetyl (Protein N-term) and MaxQuant internal normalization algorithm MaxLFQ and the search algorithm "Match between runs" were turned on. For the Phosphoproteome analysis serine, threonine and tyrosine phosphorylation (STY) was included to the variable modification. Furthermore, MaxLFQ and the search algorithm "Match between runs" remained off.

Proteomics data was further processed before analysis using the R package "DEP" (v1.18.0) [11]. Data was filtered to exclude proteins only identified by a modification site, reverse hits or possible contaminants. Additionally, proteins not detected in at least 2/3 replicates per condition were excluded. Missing data was imputed through random draws from a gaussian distribution centered around a minimal value.

Phosphoproteomics data was further processed before analysis using the R package "PhosR" (v1.6.0) [12, 13]. Data was filtered to exclude reverse hits and possible contaminants. Additionally, peptides not detected in at least 2/3 replicates per condition were excluded. Missing data was imputed through site- and condition-specific as well as tail-based imputation. Lastly, phosphoproteomics data was batch-corrected using a set of stably phosphorylated sites [12, 13] and implementation of the Removing Unwanted Variation-III method [14].

Luminex-based multiplex assay

Conditioned media (CM) for each condition and timepoint was collected from 3 dishes, yielding approx. 9 mL of CM per condition. Cells were counted at the time of CM harvesting using the Vi-CELL XR (Beckman Coulter; Software v2.03) using the settings described in Table S1. CM was centrifuged at 1500 rpm for 15 min to clear cellular debris and stored at -80 °C until use.

Protein concentrations in pg/ml were calculated and then normalized to viable cell counts to account for differences in cell density across the different conditions and timepoints.

Transwell migration assay

HMC3 cells were switched to RPMI containing 2% FCS three days before the experiment. For migration assays, HMC3 cells were seeded into the upper transwell chamber (24-well format, 8 µM pore size; cat. no. 353097, Corning) and allowed to settle for 30 min. Following, conditioned media or media with different FCS concentrations was added to the bottom well of the transwell chamber (24-well companion plate; 353504, Corning). For experiments using neutralizing antibodies, CM was incubated with neutralizing antibodies at 37 °C for 30 min before adding the mixture to the bottom well (anti-CCL2 (1 µg/ml; cat. no. MAB279, R&D systems), anti-CX3CL1 (0.5 µg/ml; cat. no. MAB3652, R&D systems), anti-CXCL10 (0.5 µg/ml; cat. no. MAB266, R&D systems), anti-CCL7 (0.2 ng/ml; cat. no. MAB282, R&D systems) and mouse IgG (2 µg/ml; cat. no. MAB002, R&D systems)). After overnight (16-18 h) incubation, cells that migrated through the transwell membrane were fixed in 4% formaldehyde, permeabilized using 0.5% Triton-X and stained using DAPI (0.5 µg/mL; cat. no. D9542, Sigma-Aldrich) before mounting the membranes on glass slides in ProLong Gold Antifade Mountant (cat. no. P10144, ThermoFisher Scientific) for imaging. The non-migrated cells in the top of the transwell chamber were removed with a cotton swap after fixation prior to permeabilization and staining. Membranes were imaged (2 technical duplicates per condition, 10-12 randomly distributed images per transwell membrane) using the Zeiss LSM 710 confocal microscope (Zeiss) and number of migrated cells was counted manually using ImageJ (v2.9.0) or automated using CellProfiler (v.4.1.3).

BT40 xenograft *in vivo* model

Six-week-old female NOD scid gamma mice (NSG), purchased from Charles River UK, were used to establish intracranial xenografts. BT-40 cells (1.5×10^5 per mouse) were injected into the forebrain using a Hamilton syringe, at the coordinates: bregma + 1 mm anterior, 1.5 mm lateral and 3 mm ventral. Bioluminescence imaging (Firefly D-Luciferin, s.c. 150mg/kg – PerkinElmer # 122799; IVIS Lumina III In Vivo Imaging System - PerkinElmer) was used to monitor intracranial

tumour growth. Treatment of animals was started for each mouse individually once bioluminescence signal reached a radiance of >10^7 photons/sec/cm^2/sr. Animals were treated daily for six days with 100 mg/kg dabrafenib (solvent: 5% DMSO, 0.5% hydroxypropylmethylcellulose, 0.2% Tween-80 in water; oral administration; MedChemExpress). Tissue samples were harvested 2 h or three days after the last dabrafenib administration for the treatment or withdrawal group respectively. Untreated control samples were isolated when bioluminescence signal reached a radiance of >10^7 photons/sec/cm^2/sr. Tissue was flash frozen and stored at -80°C until further use for subsequent RNA extraction.

Statistics

Graphical depiction was done using GraphPad Prism and R Studio using the R packages “ggplot2” (v3.4.2)[15] or “ComplexHeatmap” (v2.12.1) [16, 17].

ssGSEA analysis was performed using the ssGSEA module (version 10.1.0) [18] on GenePattern [19] using default settings except for min. gene set size which was set to 1. Gene sets related to proliferation and cell cycle were taken from the MSigDB (v7.5.1) C2 subcollection CP (Broad Institute).

Differential phosphorylation was calculated using the R package “PhosR” (v1.6.0) [12, 13].

Differential protein expression was calculated using the R package “DEP” (v1.18.0) [11]. This package was run under R version 4.1.3.

Differential gene expression was calculated using the DeSeq2 module (version 3) [20] on GenePattern [19] using default settings.

MAPK activity scores based on phosphoproteomics data were calculated using the ssGSEA module (version 10.1.0) [18] on GenePattern [19] using default settings except for min. gene set size which was set to 1. The MEK1 PTM-SEA signature, containing protein phosphorylation sites

phosphorylated by MEK1, was taken from the PTMsigDB collection (v2.0.0) [21]. ssGSEA scores were not normalized and are therefore shown as measured in arbitrary units.

The MPAS score, consisting of ten genes shown to be downregulated upon MEK inhibition, was calculated using FPKM values as previously described [22]. MPAS scores were not normalized and are therefore shown as measured in arbitrary units.

MAPK activity scores based on proteomics data were calculated using the ssGSEA module (version 10.1.0) [18] on GenePattern [19] using default settings except for min. gene set size which was set to 1. As MAPK ssGSEA score a set of proteins shown to be downregulated upon MEK inhibition was used [23]. ssGSEA scores were not normalized and are therefore shown as measured in arbitrary units.

Longitudinal k-means clustering, an adaptation of the k-means clustering method, was performed using the R package “kml” (v2.4.6) [24] with 20 iterations. Up to 20 clusters were tested and the number of clusters with the highest Calinski-Harabasz index [25] was selected. Only genes, proteins and phospho-peptides with an adjusted p-value < 0.01 for at least one timepoint were included in the analysis. Differentially regulated clusters, used for further analysis, were defined based on the cluster mean log2FC with a cut-off of ±1.5.

GO-term enrichment analysis of molecular function ontology terms was performed and visualized using ClueGo (v2.5.9)[26] in CytoScape (v3.9.1) [27]. Enrichment was calculated using the right-sided hypergeometric test, p-values were corrected using Bonferroni step down. GO-term groups were defined based on shared genes (Kappa Score: 0.4).

KSEA analysis was performed using the KSEA app using the PhosphoSitePlus and NetworkKIN kinase-substrate datasets [28–31]. For each timepoint, only phospho-peptides with a significant change relative to control were used for calculation (adjusted p-value < 0.05).

REFERENCES

1. Usta D, Sigaud R, Buhl JL, et al (2020) A cell-based MAPK reporter assay reveals synergistic MAPK pathway activity suppression by MAPK inhibitor combination in BRAF-driven pediatric low-grade glioma cells. Mol Cancer Ther 19:1736–1750. https://doi.org/10.1158/1535-7163.MCT-19-1021

2. Sigaud R, Rösch L, Gatzweiler C, et al (2022) The first-in-class ERK inhibitor ulixertinib shows promising activity in mitogen-activated protein kinase (MAPK)-driven pediatric low-grade glioma models. Neuro Oncol. https://doi.org/10.1093/neuonc/noac183

3. Jamaladdin N, Sigaud R, Kocher D, et al (2023) Key Pharmacokinetic Parameters of 74 Pediatric Anticancer Drugs Providing Assistance in Preclinical Studies. Clin Pharmacol Ther 114:904–913. https://doi.org/10.1002/cpt.3002

4. Isakoff SJ, Tabernero J, Molife LR, et al (2020) Antitumor activity of ipatasertib combined with chemotherapy: results from a phase Ib study in solid tumors. Ann Oncol 31:626–633. https://doi.org/10.1016/j.annonc.2020.02.007

5. van de Velde ME, Panetta JC, Wilhelm AJ, et al (2020) Population pharmacokinetics of vincristine related to infusion duration and peripheral neuropathy in pediatric oncology patients. Cancers (Basel) 12:1–15. https://doi.org/10.3390/cancers12071789

6. Riccardi R, Riccardi A, Lasorella A, et al (1994) Clinical pharmacokinetics of carboplatin in children. Cancer Chemother Pharmacol 33:477–483. https://doi.org/10.1007/BF00686504

7. Wishart DS, Feunang YD, Guo AC, et al (2018) DrugBank 5.0: A major update to the DrugBank database for 2018. Nucleic Acids Res 46:D1074–D1082. https://doi.org/10.1093/nar/gkx1037

8. Milde T, Oehme I, Korshunov A, et al (2010) HDAC5 and HDAC9 in medulloblastoma: Novel markers for risk stratification and role in tumor cell growth. Clin Cancer Res 16:3240–3252. https://doi.org/10.1158/1078-0432.CCR-10-0395

9. Reisinger E, Genthner L, Kerssemakers J, et al (2017) OTP: An automatized system for managing and processing NGS data. J. Biotechnol. 261:53–62

10. Cox J, Mann M (2008) MaxQuant enables high peptide identification rates, individualized p.p.b.-range mass accuracies and proteome-wide protein quantification. Nat Biotechnol 26:1367–1372. https://doi.org/10.1038/nbt.1511

11. Zhang X, Smits AH, van Tilburg GBA, et al (2018) Proteome-wide identification of ubiquitin interactions using UbIA-MS. Nat Protoc 13:530–550

12. Kim HJ, Kim T, Xiao D, Yang P (2021) Protocol for the processing and downstream

analysis of phosphoproteomic data with PhosR. STAR Protoc 2:100585. https://doi.org/10.1016/j.xpro.2021.100585

13. Kim HJ, Kim T, Hoffman NJ, et al (2021) PhosR enables processing and functional analysis of phosphoproteomic data. Cell Rep 34:108771. https://doi.org/10.1016/j.celrep.2021.108771

14. Molania R, Gagnon-Bartsch JA, Dobrovic A, Speed TP (2019) A new normalization for Nanostring nCounter gene expression data. Nucleic Acids Res 47:6073–6083. https://doi.org/10.1093/nar/gkz433

15. Wickham H (2016) ggplot2: Elegant Graphics for Data Analysis. Springer-Verlag New York

16. Gu Z (2022) Complex heatmap visualization. iMeta 1:e43. https://doi.org/10.1002/imt2.43

17. Gu Z, Eils R, Schlesner M (2016) Complex heatmaps reveal patterns and correlations in multidimensional genomic data. Bioinformatics 32:2847–2849. https://doi.org/10.1093/bioinformatics/btw313

18. Barbie DA, Tamayo P, Boehm JS, et al (2009) Systematic RNA interference reveals that oncogenic KRAS-driven cancers require TBK1. Nature 462:108–112. https://doi.org/10.1038/nature08460

19. Reich M, Liefeld T, Gould J, et al (2006) GenePattern 2.0 [2]. Nat. Genet. 38:500–501

20. Love MI, Huber W, Anders S (2014) Moderated estimation of fold change and dispersion for RNA-seq data with DESeq2. Genome Biol 15:550. https://doi.org/10.1186/s13059-014-0550-8

21. Krug K, Mertins P, Zhang B, et al (2019) A Curated Resource for Phosphosite-specific Signature Analysis. Mol Cell Proteomics 18:576–593. https://doi.org/10.1074/mcp.TIR118.000943

22. Wagle M-C, Kirouac D, Klijn C, et al (2018) A transcriptional MAPK Pathway Activity Score (MPAS) is a clinically relevant biomarker in multiple cancer types. npj Precis Oncol 2:7. https://doi.org/10.1038/s41698-018-0051-4

23. Maxwell MJ, Arnold A, Sweeney H, et al (2021) Unbiased proteomic and phosphoproteomic analysis identifies response signatures and novel susceptibilities after combined MEK and mTOR inhibition in BRAFV600E Mutant Glioma. Mol Cell Proteomics 20:. https://doi.org/10.1016/j.mcpro.2021.100123

24. Genolini C, Alacoque X, Sentenac M, Arnaud C (2015) {kml} and {kml3d}: {R} Packages to Cluster Longitudinal Data. J Stat Softw 65:1–34.

https://doi.org/10.18637/jss.v065.i04

25. Caliñski T, Harabasz J (1974) A Dendrite Method Foe Cluster Analysis. Commun Stat 3:1–27. https://doi.org/10.1080/03610927408827101

26. Bindea G, Mlecnik B, Hackl H, et al (2009) ClueGO: A Cytoscape plug-in to decipher functionally grouped gene ontology and pathway annotation networks. Bioinformatics 25:1091–1093. https://doi.org/10.1093/bioinformatics/btp101

27. Shannon P, Markiel A, Ozier O, et al (2003) Cytoscape: A software Environment for integrated models of biomolecular interaction networks. Genome Res 13:2498–2504. https://doi.org/10.1101/gr.1239303

28. Wiredja DD, Koyutürk M, Chance MR (2017) The KSEA App: a web-based tool for kinase activity inference from quantitative phosphoproteomics. Bioinformatics 33:3489–3491. https://doi.org/10.1093/bioinformatics/btx415

29. Horn H, Schoof EM, Kim J, et al (2014) KinomeXplorer: An integrated platform for kinome biology studies. Nat. Methods 11:603–604

30. Hornbeck P V., Zhang B, Murray B, et al (2015) PhosphoSitePlus, 2014: Mutations, PTMs and recalibrations. Nucleic Acids Res 43:D512–D520. https://doi.org/10.1093/nar/gku1267

31. Casado P, Rodriguez-Prados JC, Cosulich SC, et al (2013) Kinase-substrate enrichment analysis provides insights into the heterogeneity of signaling pathway activation in leukemia cells. Sci. Signal. 6
