# Supplementary material for: Rebound growth of BRAF mutant pediatric glioma cells after MAPKi withdrawal is associated with MAPK reactivation and secretion of microglia-recruiting cytokines
Source: J Neurooncol. 2024 Apr 17;168(2):317–32. doi: 10.1007/s11060-024-04672-9 (PMC11147834; doi:10.1007/s11060-024-04672-9)
Supplement: Supplementary file 2 — Supplementary Material 2 [file 11060_2024_4672_MOESM2_ESM.pdf]

Rebound growth of BRAF mutant pediatric glioma cells after MAPKi withdrawal is associated with MAPK reactivation and secretion of microglia-recruiting cytokines

Daniela Kocher[1,2,3,4], Lei Cao[5,6], Romain Guiho[5,7], Melanie Langhammer[8,9], Yun-Lu Lai[1,2,3], Pauline Becker[1,2,3,10], Hiba Hamdi[5], Dennis Friedel[4,11,12], Florian Selt[1,2,3,13], David Vonhören[4,11,12], Julia Zaman[4,11,12], Gintvile Valinciute[1,2,3], Sonja Herter[1,2,3,4], Daniel Picard[14,15,16], Johanna Rettenmeier[1,2,10,17,18], Kendra K. Maass[1,2,17,18], Kristian W. Pajtler[1,2,13,17,18], Marc Remke[19], Andreas von Deimling[11,12], Stefan Pusch[11,12], Stefan M. Pfister[1,2,13,17], Ina Oehme[1,2,3], David T.W. Jones[1,2,20], Sebastian Halbach[8,21], Tilman Brummer[8,21,22], Juan Pedro Martinez-Barbera[5], Olaf Witt[1,2,3,13], Till Milde[1,2,3,13,**], Romain Sigaud[1,2,3,*]

[1]Hopp Children's Cancer Center Heidelberg (KiTZ), Heidelberg, Germany
[2]National Center for Tumor Diseases (NCT), NCT Heidelberg, a partnership between DKFZ and Heidelberg University Hospital, Germany.
[3]German Cancer Research Center (DKFZ) Heidelberg, Clinical Cooperation Unit Pediatric Oncology, Germany
[4]Faculty of Biosciences, Heidelberg University, Heidelberg, Germany
[5]Developmental Biology and Cancer Programme, Birth Defects Research Centre, Great Ormond Street Institute of Child Health, University College London, 30 Guilford Street, London, WC1N 1EH, UK
[6]Department of Neurosurgery, Beijing Tiantan Hospital, Capital Medical University, Beijing, China
[7]Nantes Université, Oniris, INSERM, Regenerative Medicine and Skeleton, RMeS, UMR 1229, F-44000 Nantes, France
[8]Institute of Molecular Medicine and Cell Research (IMMZ), Faculty of Medicine, University of Freiburg
[9]Faculty of Biology, University of Freiburg, Freiburg, Germany
[10]Faculty of Medicine, Heidelberg University, Heidelberg, Germany.
[11]Department of Neuropathology, Heidelberg University Hospital, Heidelberg, Germany
[12]German Cancer Research Center (DKFZ), Clinical Cooperation Unit Neuropathology , Heidelberg, Germany
[13]KiTZ Clinical Trial Unit (ZIPO), Department of Pediatric Hematology and Oncology, Heidelberg University Hospital, Heidelberg, Germany
[14]Department of Pediatric Oncology, Hematology, and Clinical Immunology, Medical Faculty, and University Hospital Düsseldorf, Heinrich Heine University, Düsseldorf, Germany
[15]German Cancer Consortium (DKTK), Partner site Essen/Düsseldorf, Düsseldorf, Germany
[16]Institute of Neuropathology, Medical Faculty, and University Hospital Düsseldorf, Heinrich Heine University, Düsseldorf, Germany
[17]German Cancer Research Center (DKFZ), Division of Pediatric Neurooncology, Heidelberg, Germany
[18]Department of Pediatric Oncology, Hematology, Immunology and Pulmonology, Heidelberg University Hospital, Heidelberg, Germany
[19]Pediatric Hematology and Oncology, University Children's Hospital, Saarland University, Homburg, Germany
[20]German Cancer Research Center (DKFZ) Heidelberg, Division of Pediatric Glioma Research, Germany
[21]German Consortium for Translational Cancer Research (DKTK), Freiburg, Germany, German Cancer Research Center (DKFZ), Heidelberg, Germany
[22]Centre for Biological Signaling Studies BIOSS, University of Freiburg

*Corresponding author: Dr. Romain Sigaud, Hopp Children‘s Cancer Center Heidelberg (KiTZ), Im Neuenheimer Feld 280, 69120, Heidelberg, Germany (r.sigaud@kitz-heidelberg.de)

**Corresponding author: Prof. Dr. med. Till Milde, Hopp Children‘s Cancer Center Heidelberg (KiTZ), Im Neuenheimer Feld 280, 69120, Heidelberg, Germany (t.milde@kitz-heidelberg.de)

Fig. S1

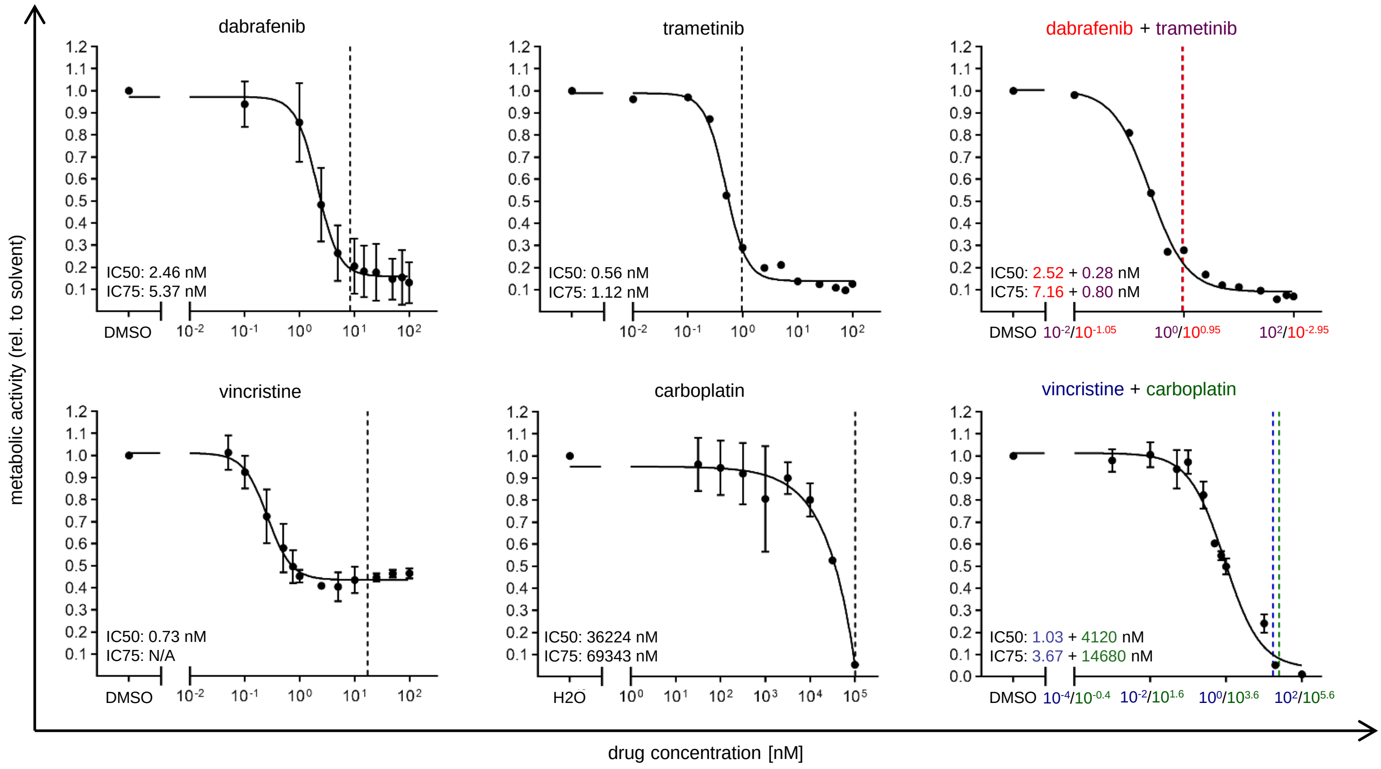

Fig. S1 Dose-response curves of BT-40 cells treated with MAPKi or chemotherapy

Metabolic activity was measured after treatment for 72h with the indicated drugs and varying concentrations. Metabolic activity is depicted relative to solvent control as mean±SD of three biological replicates. Dashed line indicates unbound Cmax concentrations. Absolute inhibitory concentrations (IC)50 and IC75 values were calculated using a 4-parameter dose-response model. N/A: not applicable

Fig. S2

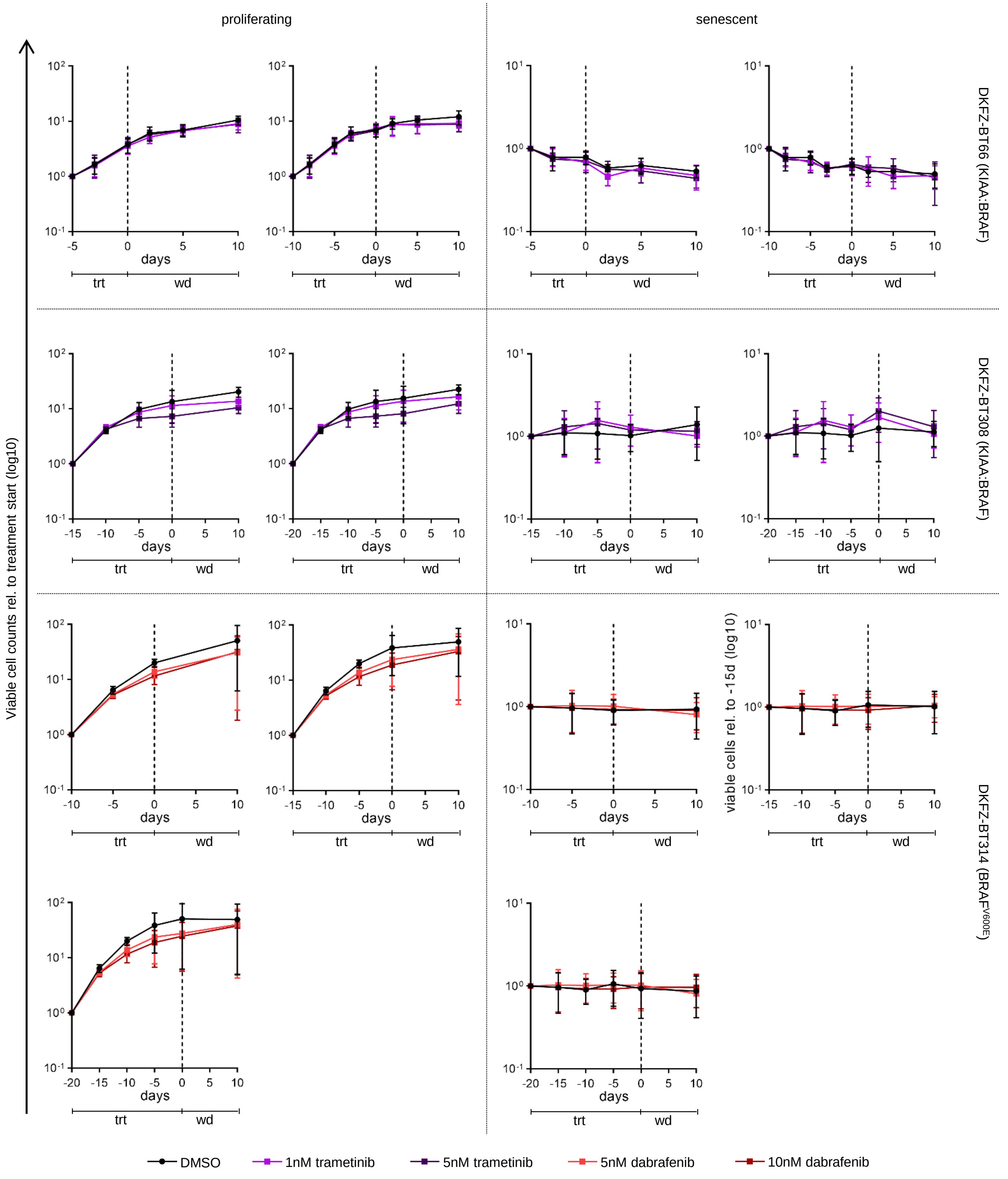

Fig. S2 Rebound growth could not be modelled in PA-derived cell lines

Viable cell counts during treatment (trt) and withdrawal (wd) using trametinib or dabrafenib. Dashed line in the graph indicates withdrawal timepoint. Viable cell counts are normalized to treatment start (first timepoint shown in each graph). Data is shown on a logarithmic scale (base 10) as mean±SD (n=3 independent biological replicates)

Fig. S3

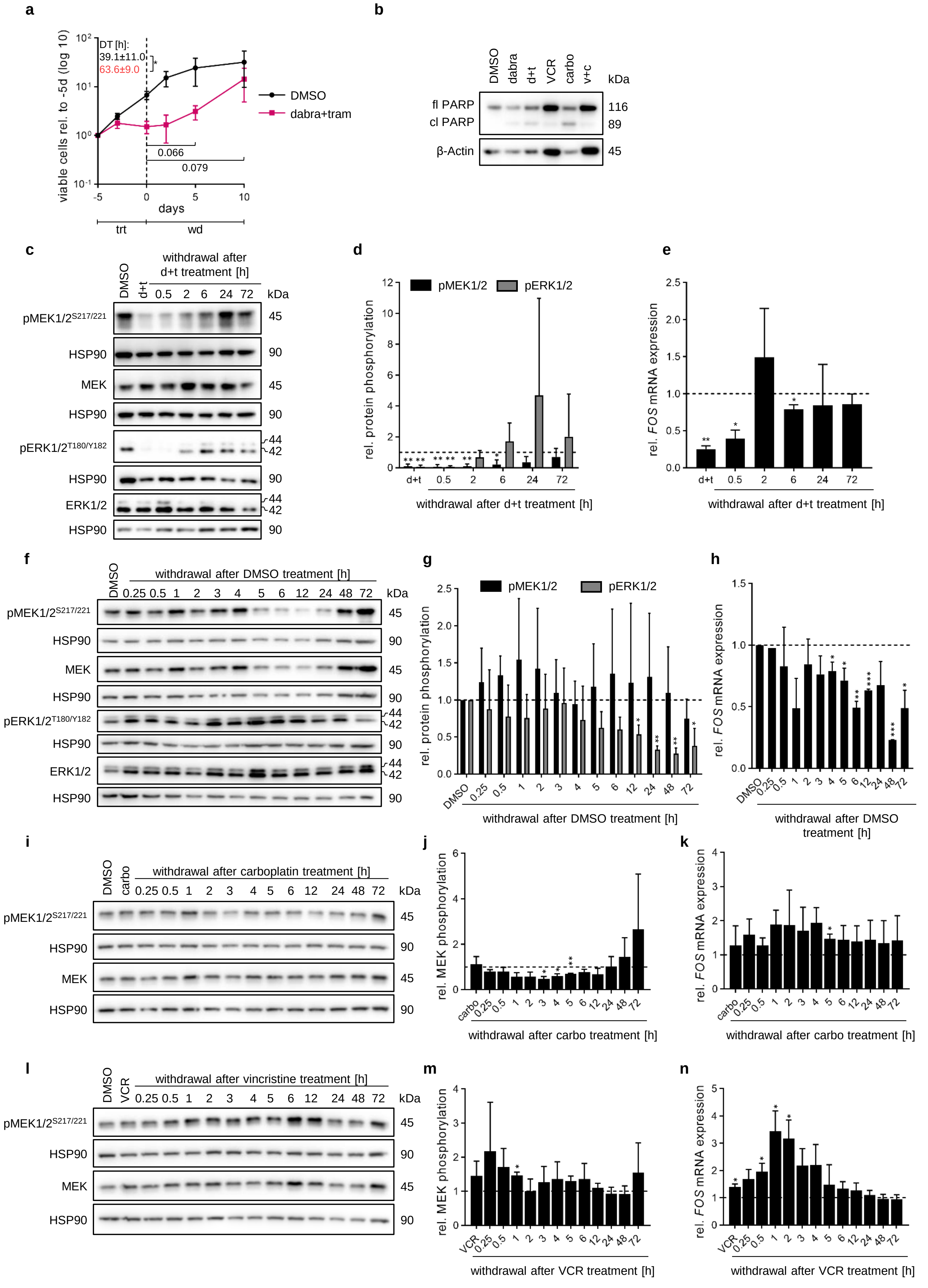
a
viable cells rel. to -5d (log 10)
DT [h]:
39.1±11.0
63.6±9.0
*
0.066
0.079
days
trt
wd
DMSO
dabra+tram
b
DMSO
dabra
d+t
VCR
carbo
v+c
kDa
fl PARP
cl PARP
β-Actin
116
89
45
c
withdrawal after d+t treatment [h]
DMSO
d+t
0.5 2 6 24 72
kDa
pMEK1/2S217/221
HSP90
MEK
pERK1/2T180/Y182
ERK1/2
45
90
44
42
d
pMEK1/2
pERK1/2
rel. protein phosphorylation
withdrawal after d+t treatment [h]
e
rel. FOS mRNA expression
withdrawal after d+t treatment [h]
f
withdrawal after DMSO treatment [h]
0.25 0.5 1 2 3 4 5 6 12 24 48 72
g
rel. protein phosphorylation
withdrawal after DMSO treatment [h]
h
rel. FOS mRNA expression
withdrawal after DMSO treatment [h]
i
carbo
withdrawal after carboplatin treatment [h]
j
rel. MEK phosphorylation
withdrawal after carbo treatment [h]
k
rel. FOS mRNA expression
withdrawal after carbo treatment [h]
l
VCR
withdrawal after vincristine treatment [h]
m
rel. MEK phosphorylation
withdrawal after VCR treatment [h]
n
rel. FOS mRNA expression
withdrawal after VCR treatment [h]

Fig. S3 Additional characterization of BT-40 rebound model

(a) Viable cell counts of BT-40 during treatment (trt) and withdrawal (wd) using 2.7 nM dabrafenib (dabra) and 0.3 nM trametinib (tram). Dashed line indicates withdrawal timepoint. Viable cell counts are normalized to treatment start (-5d). Data is shown on a logarithmic scale (base 10) as mean±SD (n=3 independent biological replicates). Unpaired two-sided t-test; ns: not significant; no indication: not tested. Doubling time (DT) was calculated from two to ten days of withdrawal for dabra+tram treatment and from minus three to five days for control for each biological replicate (n=3) and is indicated in hours as mean±SD. Unpaired two-sided t-test; * p-value ≤ 0.05

(b) Western blot analysis of full-length (fl) and cleaved (cl) PARP after treatment for five days with 5 nM dabrafenib (dabra), 2.7 nM dabrafenib and 0.3 nM trametinib (d+t), 0.75 nM vincristine (VCR), 40 µM carboplatin (carbo) or 1 nM vincristine and 4 µM carboplatin (v+c)

(c-d) Western blot analysis of MAPK activity markers after five days treatment with 2.7 nM dabrafenib and 0.3 nM trametinib (d+t) followed by treatment withdrawal. Blots shown are representative of three biological replicates (c). Quantification (d) is relative to solvent control (DMSO; dashed line) and shown as mean±SD (n=3 independent biological replicates). One-sample t-test, * p-value ≤ 0.05 ** p-value ≤ 0.01; no indication: not significant

(e) RT-qPCR analysis of FOS gene expression after five days treatment with 2.7 nM dabrafenib and 0.3 nM trametinib (d+t) followed by treatment withdrawal. Quantification is relative to DMSO control (dashed line) and shown as mean±SD (n=3 independent biological replicates). One-sample t-test, * p-value ≤ 0.05 ** p-value ≤ 0.01; no indication: not significant

(f-g) Western blot analysis of MAPK activity markers after five days treatment with DMSO (solvent control) followed by treatment withdrawal. Blots shown are representative of three biological replicates (g). Quantification (f) is relative to DMSO control (dashed line) and shown as mean±SD (n=3 independent biological replicates). One-sample t-test, * p-value ≤ 0.05 ** p-value ≤ 0.01; no indication: not significant

(h) RT-qPCR analysis of FOS gene expression after five days treatment with DMSO (solvent control) followed by treatment withdrawal. Quantification is relative to DMSO control (dashed line) and shown as mean±SD (n=3 independent biological replicates). One-sample t-test, * p-value ≤ 0.05 ** p-value ≤ 0.01, *** p-value ≤ 0.001; no indication: not significant

(i-j) Western blot analysis of MEK phosphorylation after five days treatment with 40 µM carboplatin (carbo) followed by treatment withdrawal. Blots shown are representative of three biological replicates (i). Quantification (j) is relative to DMSO control (dashed line) and shown as mean±SD (n=3 independent biological replicates). One-sample t-test, * p-value ≤ 0.05 ** p-value ≤ 0.01; no indication: not significant

(k) RT-qPCR analysis of FOS gene expression after five days treatment with 40 µM carboplatin (carbo) followed by treatment withdrawal. Quantification is relative to DMSO control (dashed line) and shown as mean±SD (n=3 independent biological replicates). One-sample t-test, * p-value ≤ 0.05; no indication: not significant

(l-m) Western blot analysis of MEK phosphorylation after five days treatment with 0.75 nM vincristine (VCR) followed by treatment withdrawal. Blots shown are representative of three biological replicates (l). Quantification (m) is relative to DMSO control (dashed line) and shown as mean±SD (n=3 independent biological replicates). One-sample t-test, * p-value ≤ 0.05; no indication: not significant

(n) RT-qPCR analysis of FOS gene expression after five days treatment with 0.75 nM vincristine (VCR) followed by treatment withdrawal. Quantification is relative to DMSO control (dashed line) and shown as mean±SD (n=3 independent biological replicates). One-sample t-test, * p-value ≤ 0.05; no indication: not significant

Fig. S4

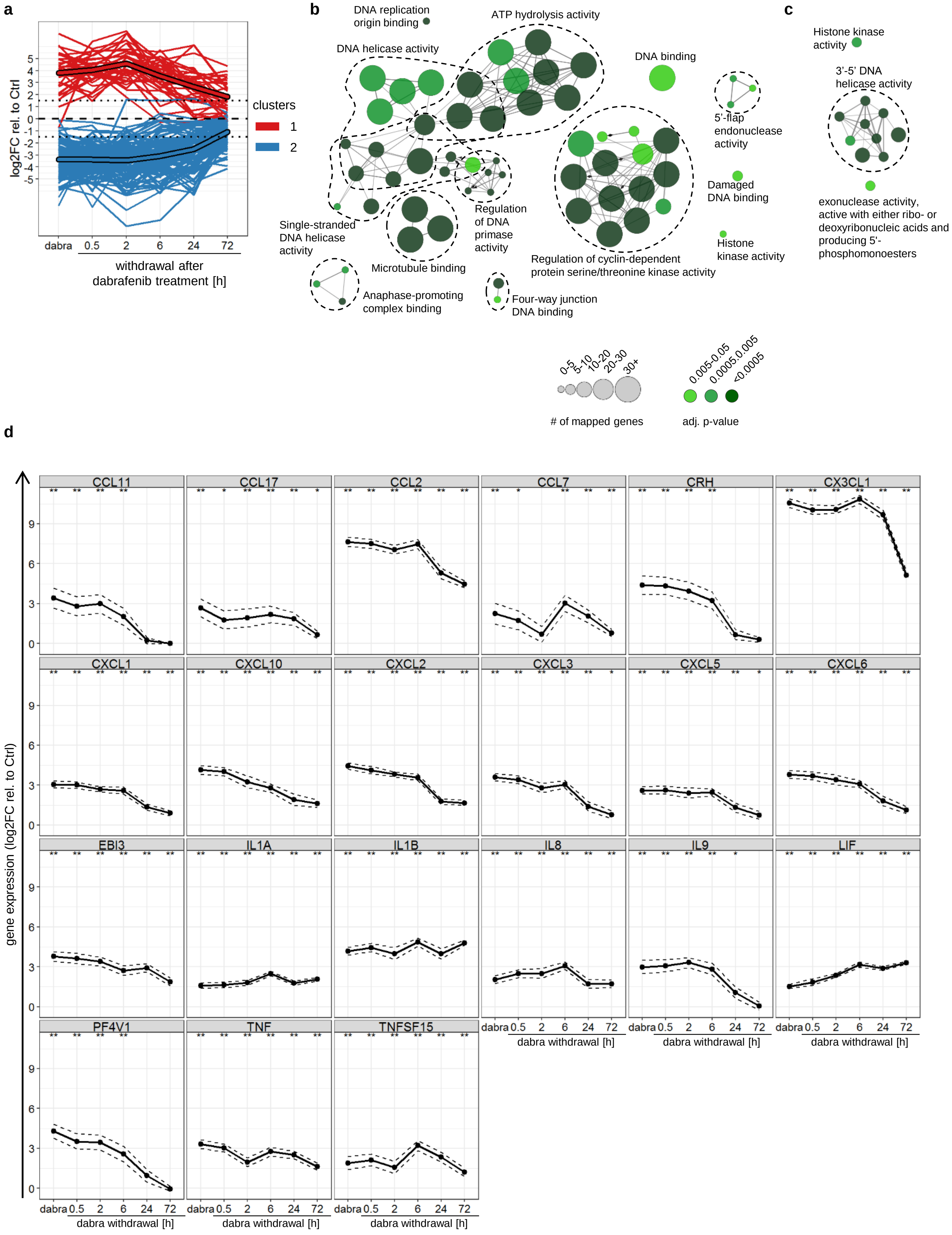
a
b
c
d
log2FC rel. to Ctrl
clusters
1
2
dabra
0.5
2
6
24
72
withdrawal after dabrafenib treatment [h]
DNA replication origin binding
ATP hydrolysis activity
DNA helicase activity
DNA binding
5'-flap endonuclease activity
Damaged DNA binding
Histone kinase activity
Single-stranded DNA helicase activity
Regulation of DNA primase activity
Microtubule binding
Regulation of cyclin-dependent protein serine/threonine kinase activity
Anaphase-promoting complex binding
Four-way junction DNA binding
3'-5' DNA helicase activity
exonuclease activity, active with either ribo- or deoxyribonucleic acids and producing 5'-phosphomonoesters
0-5
5-10
10-20
20-30
30+
# of mapped genes
0.005-0.05
0.0005-0.005
<0.0005
adj. p-value
gene expression (log2FC rel. to Ctrl)
CCL11
CCL17
CCL2
CCL7
CRH
CX3CL1
CXCL1
CXCL10
CXCL2
CXCL3
CXCL5
CXCL6
EBI3
IL1A
IL1B
IL8
IL9
LIF
PF4V1
TNF
TNFSF15
dabra withdrawal [h]

Fig. S4 Multi-omics analysis of dabrafenib treatment and withdrawal in BT-40

(a) Longitudinal k-means clustering of differentially regulated phosphopeptides after five days treatment with 5 nM dabrafenib (dabra) followed by withdrawal relative to solvent control (five days DMSO; dashed line). Only phosphopeptides with an adjusted p-value < 0.01 for at least one timepoint were included in the analysis. Unframed lines represent single phosphopeptides, framed lines show cluster means. Dotted line: log2FC = +/-1.5. n=3 independent biological replicates

(b-c) GO-term enrichment analysis of downregulated genes, clusters 4 and 5 from Fig. 2d (cluster mean ≤ -1.5), (b) and proteins, cluster 2 from Fig. 2e (cluster mean ≤ -1.5), (c). Only terms with significant enrichment (adj. p-value ≤ 0.05) are shown. GO-term groups are defined by overlapping genes and named based on the GO-Term with highest percentage of mapped genes

(d) Cytokine gene expression, determined by RNAseq, during treatment for five days with 5 nM dabrafenib followed by withdrawal. Data is shown as log2FC relative to solvent control (five days DMSO) as mean±SE (n=3 independent biological replicates). Wald test, Benjamini-Hochberg p-value correction, * adj-p-value ≤ 0.05 ** adj-p-value ≤ 0.01 *** adj-p-value ≤ 0.01; no indication: not significant

Fig. S5

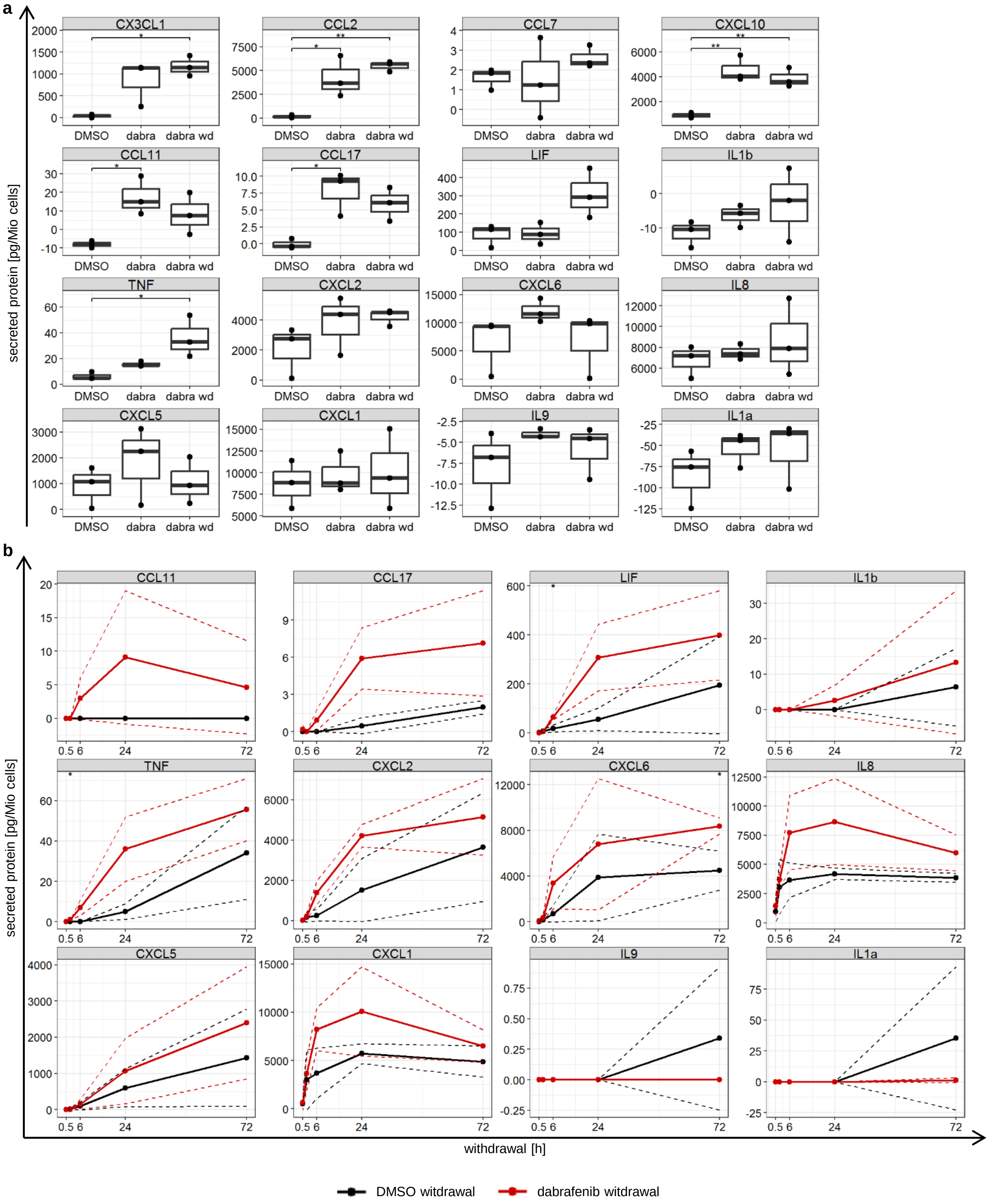

a
secreted protein [pg/Mio cells]
CX3CL1
CCL2
CCL7
CXCL10
CCL11
CCL17
LIF
IL1b
TNF
CXCL2
CXCL6
IL8
CXCL5
CXCL1
IL9
IL1a
DMSO
dabra
dabra wd
b
secreted protein [pg/Mio cells]
CCL11
CCL17
LIF
IL1b
TNF
CXCL2
CXCL6
IL8
CXCL5
CXCL1
IL9
IL1a
withdrawal [h]
DMSO witdrawal
dabrafenib witdrawal

Fig. S5 Cytokine secretion during dabrafenib treatment and withdrawal in BT-40

(a) Multiplexed ELISA assay results showing cytokine secretion in BT-40 treated for five days with DMSO (solvent control) or 5 nM dabrafenib (dabra) and 24 h after dabrafenib withdrawal (dabra wd). For all conditions, conditioned media was collected 24 h after the last medium change. Boxplots depict the median, first and third quartiles. Whiskers extend from the hinge to the largest/smallest value no further than 1.5 * IQR from the hinge (where IQR is the interquartile range) n=3 independent biological replicates. One-way ANNOVA, Tukey post-hoc test, * adj-p-value ≤ 0.05 ** adj-p-value ≤ 0.01 ** adj-p-value ≤ 0.01; no indication: not significant

(b) Multiplexed ELISA assay showing cytokine secretion during withdrawal after treatment for five days with either DMSO (solvent control) or 5 nM dabrafenib. Data for 24 h dabrafenib withdrawal is the same as shown in panel a. Data is shown as mean±SD (n=3 independent biological replicates). Two-tailed unpaired t-test, * p-value ≤ 0.05 ** p-value ≤ 0.01; not indicated: not significant

Fig. S6

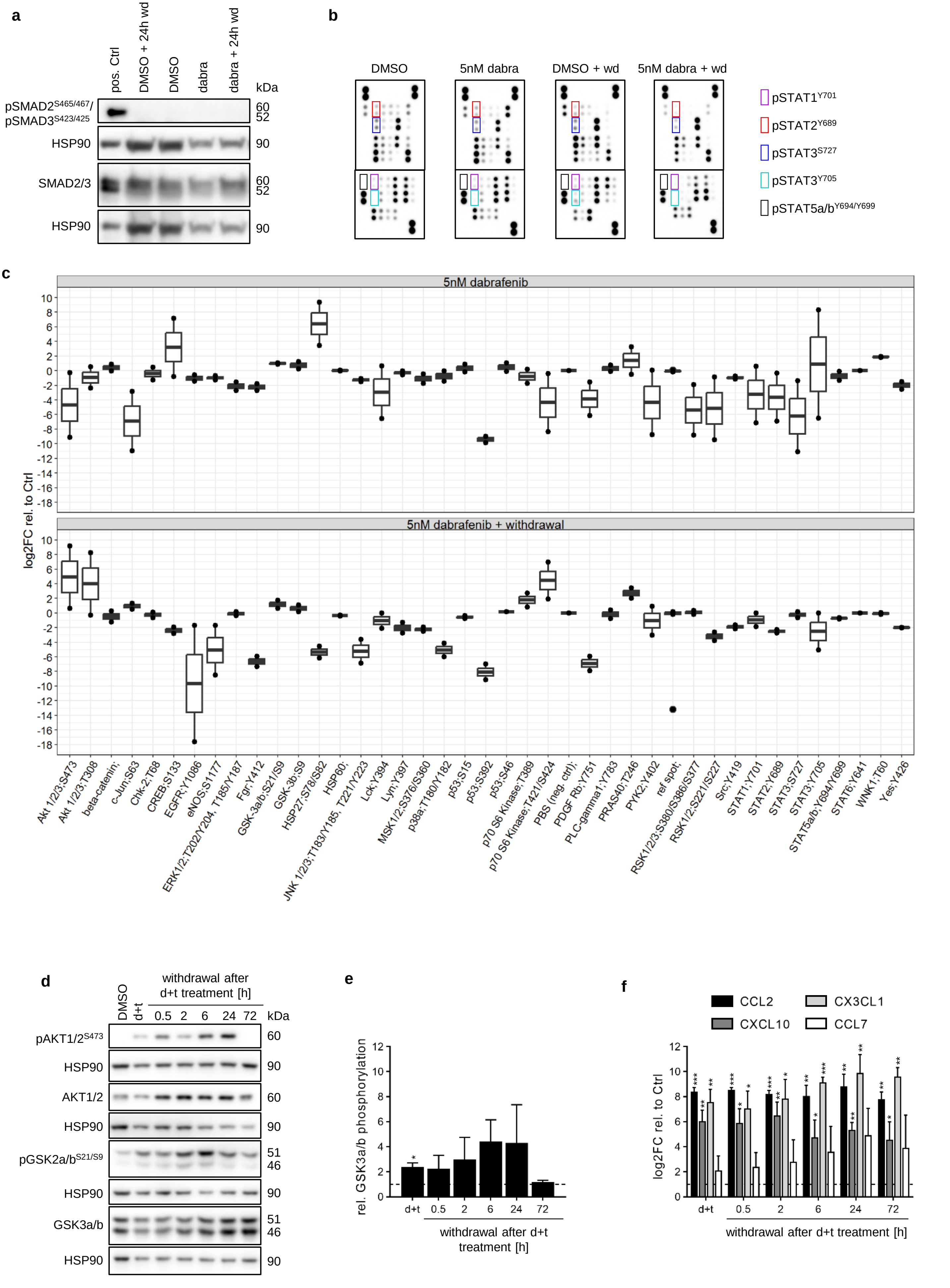
a
pos. Ctrl
DMSO + 24h wd
DMSO
dabra
dabra + 24h wd
kDa
pSMAD2S465/467/
pSMAD3S423/425
HSP90
SMAD2/3
HSP90
60
52
90
b
DMSO
5nM dabra
DMSO + wd
5nM dabra + wd
pSTAT1Y701
pSTAT2Y689
pSTAT3S727
pSTAT3Y705
pSTAT5a/bY694/Y699
c
5nM dabrafenib
5nM dabrafenib + withdrawal
log2FC rel. to Ctrl
Akt 1/2/3;S473
Akt 1/2/3;T308
beta-catenin;
c-Jun;S63
Chk-2;T68
CREB;S133
EGFR;Y1086
eNOS;S1177
ERK1/2;T202/Y204, T185/Y187
Fgr;Y412
GSK-3a/b;S21/S9
GSK-3b;S9
HSP27;S78/S82
HSP60;
JNK 1/2/3;T183/Y185, T221/Y223
Lck;Y394
Lyn;Y397
MSK1/2;S376/S360
p38a;T180/Y182
p53;S15
p53;S392
p53;S46
p70 S6 Kinase;T389
p70 S6 Kinase;T421/S424
PBS (neg. ctrl);
PDGF Rb;Y751
PLC-gamma1;Y783
PRAS40;T246
PYK2;Y402
ref spot;
RSK1/2/3;S380/S386/S377
RSK1/2;S221/S227
Src;Y419
STAT1;Y701
STAT2;Y689
STAT3;S727
STAT3;Y705
STAT5a/b;Y694/Y699
STAT6;Y641
WNK1;T60
Yes;Y426
d
DMSO
d+t
withdrawal after d+t treatment [h]
0.5
2
6
24
72
kDa
pAKT1/2S473
HSP90
AKT1/2
HSP90
pGSK2a/bS21/S9
HSP90
GSK3a/b
HSP90
e
rel. GSK3a/b phosphorylation
withdrawal after d+t treatment [h]
f
CCL2
CX3CL1
CXCL10
CCL7
log2FC rel. to Ctrl
withdrawal after d+t treatment [h]

Fig. S6 Validation of multi-omics results

(a) Western blot analysis of SMAD2/3 phosphorylation during five days treatment with either DMSO (solvent control) or 5 nM dabrafenib (dabra), followed by 24 h withdrawal (wd). Cell extract of HT-1080 treated with 10 ng/mL TGF-beta for 30 min was used as a positive control (pos. Ctrl). Blots shown are representative of three independent biological replicates.

(b-c) Kinase phosphorylation array of samples treated for five days with 5 nM dabrafenib or DMSO (solvent control) followed by 24 h withdrawal (wd). Arrays (b) consist of two membranes, each target is detected in duplicates. Arrays shown are representative of two biological replicates. Arrays shown are the same as in Fig. 2j but at a higher exposure time. Quantification (c) is shown as log2FC relative to the respective DMSO control. Boxplots depict the median, first and third quartiles. Whiskers extend from the hinge to the largest/smallest value no further than 1.5 * IQR from the hinge (where IQR is the interquartile range) n=2 independent biological replicates

(d-e) Western blot analysis of AKT activity markers after five days treatment with 2.7 nM dabrafenib and 0.3 nM trametinib (d+t) followed by treatment withdrawal. Blots shown are representative of three independent biological replicates (d). Quantification (e) is relative to solvent control (DMSO; dashed line) and shown as mean±SD (n=3 independent biological replicates). One-sample t-test, p-value ≤ 0.05 ** p-value ≤ 0.01 *** p-value ≤ 0.01; no indication: not significant

(f) RT-qPCR analysis of cytokine gene expression after five days treatment with 2.7 nM dabrafenib and 0.3 nM trametinib (d+t) followed by treatment withdrawal. Quantification is relative to solvent control (DMSO; dashed line) and shown as mean±SD (n=3 independent biological replicates). CCL7 was undetected in some samples, for these samples Ct values were set to Ctmax plus 0.5. One-sample t-test, p-value ≤ 0.05 ** p-value ≤ 0.01 *** p-value ≤ 0.01; no indication: not significant

Fig. S7

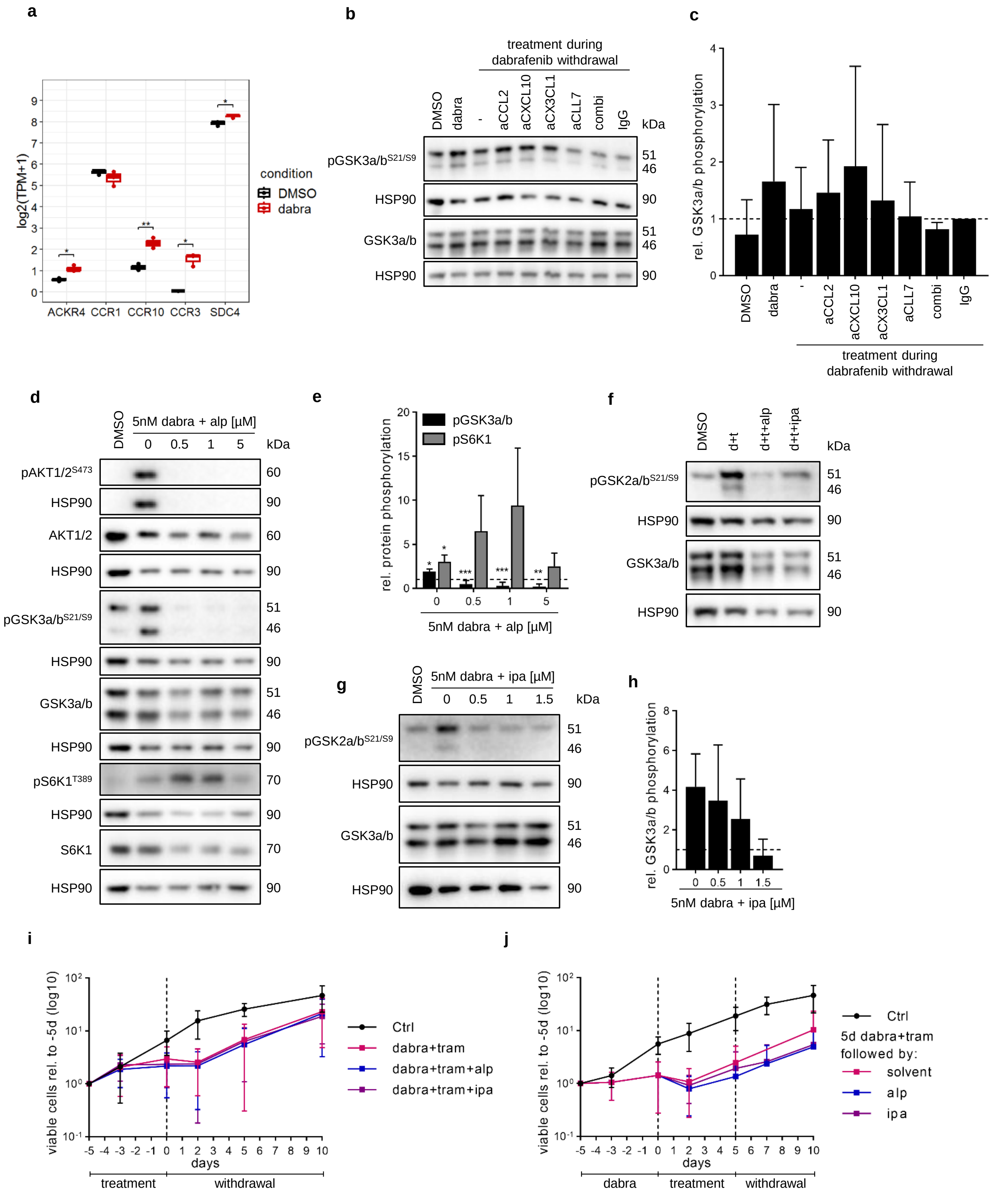
a
log2(TPM+1)
ACKR4
CCR1
CCR10
CCR3
SDC4
condition
DMSO
dabra
b
treatment during dabrafenib withdrawal
DMSO
dabra
aCCL2
aCXCL10
aCX3CL1
aCLL7
combi
IgG
kDa
pGSK3a/b S21/S9
HSP90
GSK3a/b
c
rel. GSK3a/b phosphorylation
d
5nM dabra + alp [µM]
pAKT1/2 S473
AKT1/2
pS6K1 T389
S6K1
e
pGSK3a/b
pS6K1
rel. protein phosphorylation
f
d+t
d+t+alp
d+t+ipa
pGSK2a/b S21/S9
g
5nM dabra + ipa [µM]
h
i
viable cells rel. to -5d (log10)
days
Ctrl
dabra+tram
dabra+tram+alp
dabra+tram+ipa
treatment
withdrawal
j
5d dabra+tram followed by:
solvent
alp
ipa
dabra

Fig. S7 Inhibition of cytokines and AKT signaling in BT-40

(a) Cytokine receptor gene expression in log2(TPM+1), determined by RNAseq, during treatment for five days with DMSO or 5 nM dabrafenib. Boxplots depict the median, first and third quartiles. Whiskers extend from the hinge to the largest/smallest value no further than 1.5 * IQR from the hinge (where IQR is the interquartile range) (n=3 independent biological replicates). Two-tailed unpaired t-test, p-value ≤ 0.05 ** p-value ≤ 0.01 *** p-value ≤ 0.01; no indication: not significant

(b-c) Western blot analysis of GSK3a/b phosphorylation after five days treatment with 5 nM dabrafenib followed by 24 h of withdrawal (-) or treatment with antibodies neutralizing CCL2 (aCCL2, 0.5 µg/mL), CX3CL1 (aCX3CL1, 0.25 µg/mL), CXCL10 (aCXCL10, 0.25 µg/mL) and CCL7 (aCCL7, 0.1 ng/mL) individually or as combination (combi). Blots shown are representative of three independent biological replicates (b). Quantification (c) is relative to IgG control (IgG, 1 µg/mL). One-sample t-test; no indication: not significant

(d-e) Western blot analysis of AKT activity markers after five days treatment with 5 nM dabrafenib (dabra) alone or in combination with alpelisib (alp). Blots shown are representative of three independent biological replicates (d). Quantification (e) is relative to solvent control (DMSO; dashed line) and shown as mean±SD. One-sample t-test, p-value ≤ 0.05 ** p-value ≤ 0.01 *** p-value ≤ 0.01

(f) Western blot analysis of GSK3a/b phosphorylation after five days treatment with 2.7 nM dabrafenib and 0.3 nM trametinib (d+t) alone or in combination with either 5 µM alpelisib (alp) or 1 µM ipatasertib (ipa). Blots shown are representative of three independent biological replicates

(g-h) Western blot analysis of GSK3a/b phosphorylation after five days treatment with 5 nM dabrafenib (dabra) alone or in combination with ipatasertib (ipa). Blots shown are representative of three independent biological replicates (g). Quantification (h) is relative to solvent control (DMSO; dashed line) and shown as mean±SD. One-sample t-test; no indication: not significant

(i) Viable cell counts during treatment with 2.7 nM dabrafenib and 0.3 nM trametinib (dabra+tram) alone or in combination 5 µM alpelisib (alp) or 1 µM ipatasertib (ipa) followed by ten days of withdrawal. Dashed line indicates withdrawal timepoint. Viable cell counts are normalized to treatment start (-5d). Data is shown on a logarithmic scale (base 10) as mean±SD of three biological replicates

(j) Viable cell counts during treatment with 2.7 nM dabrafenib and 0.3 nM trametinib (dabra+tram) followed by withdrawal. During withdrawal cells were either untreated (=solvent) or treated for five days with 5 µM alpelisib (alp) or 1 µM ipatasertib (ipa), followed by five days of withdrawal. Dashed lines indicate withdrawal timepoints. Viable cell counts are normalized to treatment start (-5d). Data is shown on a logarithmic scale (base 10) as mean±SD of three biological replicates

Fig. S8

a

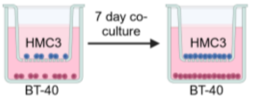

b

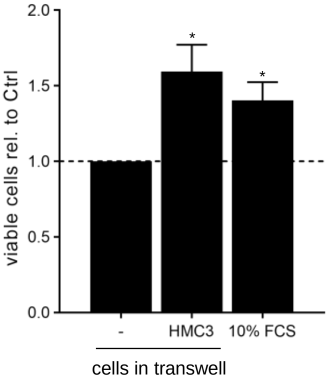

Fig. S8 BT-40 – HMC3 transwell co-culture

(a) Schematic of transwell co-culture setup

(b) Viable cell counts of BT-40 after seven days with or without (-) HMC3 co-culture. BT-40 cultured in 10% FCS were used as pos. control. Viable cell counts are shown as mean±SD (n=3 independent biological replicates) relative to control (-). One-sample t-test, * p-value ≤ 0.05

Fig. S9

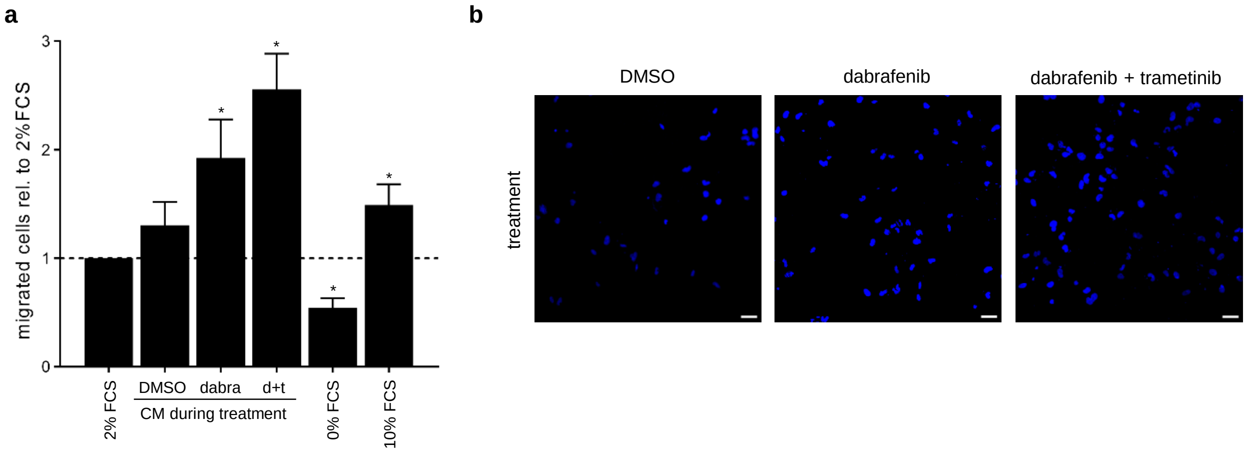

Fig. S9 HMC3 transwell migration using conditioned media collected during MAPKi treatment

(a) Transwell migration assay of HMC3 cells towards conditioned media (CM) collected from BT-40 cells after after five days treatment with DMSO (solvent control), 5nM dabrafenib (d) or 2.7 nM dabrafenib and 0.3 nM trametinib (d+t). 2% FCS serves as baseline control as CM contains 2% FCS, 0% FCS as negative control and 10% FCS as positive control. Quantification is shown as mean±SD (n=3 independent biological replicates; 2 technical duplicates per condition; 10-12 randomly distributed images were quantified per transwell) relative to 2% FCS. One-sample t-test, * p-value ≤ 0.05; no indication: not significant

(b) Representative fluorescence images showing HMC3 migrated through the transwell, nuclei were stained with DAPI. Representative images for FCS controls are shown in Fig. 5D. Scale bar = 50 µM
