# Supplementary material for: Rebound growth of BRAF mutant pediatric glioma cells after MAPKi withdrawal is associated with MAPK reactivation and secretion of microglia-recruiting cytokines
Source: J Neurooncol. 2024 Apr 17;168(2):317–32. doi: 10.1007/s11060-024-04672-9 (PMC11147834; doi:10.1007/s11060-024-04672-9)
Supplement: Supplementary file 3 — Supplementary Material 3 [file 11060_2024_4672_MOESM3_ESM.pdf]

Rebound growth of BRAF mutant pediatric glioma cells after MAPKi withdrawal is associated with MAPK reactivation and secretion of microglia-recruiting cytokines

Daniela Kocher[1,2,3,4], Lei Cao[5,6], Romain Guiho[5,7], Melanie Langhammer[8,9], Yun-Lu Lai[1,2,3], Pauline Becker[1,2,3,10], Hiba Hamdi[5], Dennis Friedel[4,11,12], Florian Selt[1,2,3,13], David Vonhören[4,11,12], Julia Zaman[4,11,12], Gintvile Valinciute[1,2,3], Sonja Herter[1,2,3,4], Daniel Picard[14,15,16], Johanna Rettenmeier[1,2,10,17,18], Kendra K. Maass[1,2,17,18], Kristian W. Pajtler[1,2,13,17,18], Marc Remke[19], Andreas von Deimling[11,12], Stefan Pusch[11,12], Stefan M. Pfister[1,2,13,17], Ina Oehme[1,2,3], David T.W. Jones[1,2,20], Sebastian Halbach[8,21], Tilman Brummer[8,21,22], Juan Pedro Martinez-Barbera[5], Olaf Witt[1,2,3,13], Till Milde[1,2,3,13,**], Romain Sigaud[1,2,3,*]

[1]Hopp Children's Cancer Center Heidelberg (KiTZ), Heidelberg, Germany
[2]National Center for Tumor Diseases (NCT), NCT Heidelberg, a partnership between DKFZ and Heidelberg University Hospital, Germany.
[3]German Cancer Research Center (DKFZ) Heidelberg, Clinical Cooperation Unit Pediatric Oncology, Germany
[4]Faculty of Biosciences, Heidelberg University, Heidelberg, Germany
[5]Developmental Biology and Cancer Programme, Birth Defects Research Centre, Great Ormond Street Institute of Child Health, University College London, 30 Guilford Street, London, WC1N 1EH, UK
[6]Department of Neurosurgery, Beijing Tiantan Hospital, Capital Medical University, Beijing, China
[7]Nantes Université, Oniris, INSERM, Regenerative Medicine and Skeleton, RMeS, UMR 1229, F-44000 Nantes, France
[8]Institute of Molecular Medicine and Cell Research (IMMZ), Faculty of Medicine, University of Freiburg
[9]Faculty of Biology, University of Freiburg, Freiburg, Germany
[10]Faculty of Medicine, Heidelberg University, Heidelberg, Germany.
[11]Department of Neuropathology, Heidelberg University Hospital, Heidelberg, Germany
[12]German Cancer Research Center (DKFZ), Clinical Cooperation Unit Neuropathology , Heidelberg, Germany
[13]KiTZ Clinical Trial Unit (ZIPO), Department of Pediatric Hematology and Oncology, Heidelberg University Hospital, Heidelberg, Germany
[14]Department of Pediatric Oncology, Hematology, and Clinical Immunology, Medical Faculty, and University Hospital Düsseldorf, Heinrich Heine University, Düsseldorf, Germany
[15]German Cancer Consortium (DKTK), Partner site Essen/Düsseldorf, Düsseldorf, Germany
[16]Institute of Neuropathology, Medical Faculty, and University Hospital Düsseldorf, Heinrich Heine University, Düsseldorf, Germany
[17]German Cancer Research Center (DKFZ), Division of Pediatric Neurooncology, Heidelberg, Germany
[18]Department of Pediatric Oncology, Hematology, Immunology and Pulmonology, Heidelberg University Hospital, Heidelberg, Germany
[19]Pediatric Hematology and Oncology, University Children's Hospital, Saarland University, Homburg, Germany
[20]German Cancer Research Center (DKFZ) Heidelberg, Division of Pediatric Glioma Research, Germany
[21]German Consortium for Translational Cancer Research (DKTK), Freiburg, Germany, German Cancer Research Center (DKFZ), Heidelberg, Germany
[22]Centre for Biological Signaling Studies BIOSS, University of Freiburg

*Corresponding author: Dr. Romain Sigaud, Hopp Children‘s Cancer Center Heidelberg (KiTZ), Im Neuenheimer Feld 280, 69120, Heidelberg, Germany (r.sigaud@kitz-heidelberg.de)

**Corresponding author: Prof. Dr. med. Till Milde, Hopp Children‘s Cancer Center Heidelberg (KiTZ), Im Neuenheimer Feld 280, 69120, Heidelberg, Germany (t.milde@kitz-heidelberg.de)

SUPPLEMENTARY TABLES

Table S1 Vi-CELL XR counting settings

Cell brightness	85%
Cell sharpness	100
Viable cell spot brightness	65%
Viable cell spot area	5%
Minimum circularity	0
Decluster degree	Medium
Minimum diameter	5 microns
Maximum diameter	50 microns
Images	50
Aspirate cycles	1
Trypan blue mixing cycles	3

Table S2 Seeding densities

wd = withdrawal

Experiment	Format	Cell line	Condition	Seeding density (number of cells)
Metabolic activity assay	96-well-plate	BT-40		$0.03x10^6$
Cell counting for growth curve analysis	6-well-plate	BT-40		$0.2x10^6$
		DKFZ-BT66 proliferating		$0.1x10^6$
		DKFZ-BT66 senescent		$0.2x10^6$
		DKFZ-BT308 and DKFZ-BT314 senescent and proliferating		$0.1x10^6$
Sample generation for qPCR and WB analysis	6-well-plate	BT-40	5 days DMSO + wd	$0.2x10^6$
			5 days treatment + wd	$1x10^6$
RNAseq sample generation	6 cm dish	BT-40	5 days DMSO + wd	$0.6x10^6$
			5 days dabrafenib + wd	$1.5x10^6$
Sample generation for LC-MS/MS proteomics and phosphoproteomics	10 cm dish (3 per sample)	BT40	5 days DMSO + wd	$2x10^6$
			5 days dabrafenib + wd	$5x10^6$
Multiplexed ELISA assay	6 cm dish	BT-40	5 days DMSO + wd	$0.4x10^6$
			5 days dabrafenib + wd	$1.5x10^6$
	6 cm dish	BT-40	5 days DMSO + wd	$0.4x10^6$

Kinase phosphorylation array			5 days dabrafenib + wd	$1.5x10^6$
Conditioned media collection	6 cm dish	BT-40	5 days DMSO + wd	$0.4x10^6$
			5 days dabrafenib + wd	$1.5x10^6$
Transwell migration assay	24-well transwells	HMC3		$0.1x10^6$
Transwell co-culture	6-well-plate	BT-40		$0.2x10^6$
	6-well transwells	HMC3		$0.08x10^6$

Table S3 Inhibitors used in *in vitro* experiments

Inhibitor	Stock concentration [mM]	Solvent	Supplier
Dabrafenib	10	DMSO	Selleckchem
Trametinib	10	DMSO	Selleckchem
Vincristine	10	DMSO	Selleckchem
Carboplatin	27	Saline solution	Pharmacy of the university hospital of Heidelberg
Alpelisip	10	DMSO	MedChemExpress
Ipatasertib	10	DMSO	MedChemExpress

Table S4 Pharmacokinetic data for inhibitors used

Plasma-protein-binding was taken from DrugBank (PMID: 29126136).

**Inhibitor**	**C_{max} [nM]**	**Plasma-protein-binding (%)**	**Unbound C_{max} [nM]**	**Reference for C_{max}**
Dabrafenib	2856.2	99.7	8.57	PMID: 37441736
Trametinib	36.07	97.4	0.94	PMID: 37441736
Vincristine	69.43	75	17.36	PMID: 32635465
Carboplatin	59000	25-50	29500-44250	PMID: 8137457
Alpelisip	7243.87	89	796.83	PMID: 37441736
Ipatasertib	1065.5	No information found	N/A	PMID: 32205017

Table S5 Primers

Gene	Forward primer 5'-3'	Reverse primer 5'-3'	Supplier
ACTB	CTGGAACGGTGAAGGTGACA	AAGGGACTTCCTGTAACAATGCA	Invitrogen
TBP	AGAACAACAGCCTGCCAC	GTTGCTCTTCCAAAATAGACAGAC	Invitrogen
FOS	/	/	Qiagen (Hs_FOS_1_SG, cat. no. 249900)
CCL2 (PMID: 30504064)	GCTCAGCCAGATGCAATCAAT	ACTTGCTGCTGGTGATTCTTCTA	Invitrogen
CXCL10 (PMID: 18495678)	GAACTGTACGCTGTACCTGCA	TTGATGGCCTTCGATTCTGGA	Invitrogen
CX3CL1 (PMID: 26015616)	GGATGCAGCCTCACAGTCCTTAC	GGCCTCAGGGTCCAAAGACA	Invitrogen
CCL7 (PMID: 33257678)	GTCCCCGGGAAGCTGTAATC	GCTTTGGAGTTTGGGTTTTCTT	Invitrogen

Table S6 Antibodies and pos. control used for WB analysis

Antibody	Dilution	Source/clonality	Supplier	Catalog number
pMEK1/2 (S217/S221)	1:1000	Rabbit monoclonal	Cell Signaling	9154T
MEK1/2	1:1000	Rabbit polyclonal	Cell Signaling	9122S
pERK1/2 (T202/T204)	1:1000	Rabbit monoclonal	Cell Signaling	4377S
ERK1/2	1:1000	Rabbit monoclonal	Cell Signaling	4695P
pFRA1 (S265)	1:1000	Rabbit monoclonal	Cell Signaling	5841S
FRA1	1:1000	Rabbit monoclonal	Cell Signaling	5281S
DUSP6	1:1000	Rabbit polyclonal	Cell Signaling	39441
pAKT1/2 (S473)	1:1000	Rabbit polyclonal	Cell Signaling	9271
AKT1/2	1:1000	Rabbit polyclonal	Cell Signaling	9272
pGSK3a/b (S21/S9)	1:1000	Rabbit polyclonal	Cell Signaling	9331S
GSK3a/b	1:1000	Rabbit monoclonal	Cell Signaling	5676T
pS6K1	1:1000	Rabbit polyclonal	Sigma-Aldrich	07-018
S6K1	1:1000	Rabbit polyclonal	Cell Signaling	9202S
PARP	1:500	Rabbit monoclonal	Cell Signaling	9532S
pSMAD2 (S465/467)/ pSMAD3 (S423/425)	1:1000	Rabbit monoclonal	Cell Signaling	8828S
SMAD2/3	1:1000	Rabbit monoclonal	Cell Signaling	8685T
β-Actin	1:10000	Mouse monoclonal	Sigma- Aldrich	A5441
HSP90	1:5000	Rabbit polyclonal	Cell Signaling	4874
goat-anti-mouse IgG HRP	1:10000	polyclonal	Dianova	115-035-003
donkey-anti-rabbit IgG HRP	1:10000	polyclonal	Promega	V795A
SMAD2/3 controls			Cell Signaling	12052S

Table S7 Custom detection antibody multiplexes used for BioPlex assay

Target	Cat. no.	Standard used	Multiplex
IL-1B	171B5001M	Bio-Plex Pro HuCSP standard	1
IL-8	171B5008M	Bio-Plex Pro HuCSP standard	1
CXCL1	171B6007M	Bio-Plex Pro HuCSP standard	1
CCL11	171B5015M	Bio-Plex Pro HuCSP standard	1
CCL2	171B5021M	Bio-Plex Pro HuCSP standard	1
IL-9	171B5009M	Bio-Plex Pro HuCSP standard	1
CXCL10	171B5020M	Bio-Plex Pro HuCSP standard	1
IL-1A	171B6001M	Bio-Plex Pro HuCSP standard	1
LIF	171B6011M	Bio-Plex Pro HuCSP standard	1
CXCL5	171BK14MR2	Bio-Plex Pro Human Chemokine standard	2
CXCL6	171BK19MR2	Bio-Plex Pro Human Chemokine standard	2
CCL7	171BK38MR2	Bio-Plex Pro Human Chemokine standard	2
TNF-alpha	171BK55MR2	Bio-Plex Pro Human Chemokine standard	2
CX3CL1	171BK18MR2	Bio-Plex Pro Human Chemokine standard	2
CXCL2	171BK23MR2	Bio-Plex Pro Human Chemokine standard	2
CCL17	171BK53MR2	Bio-Plex Pro Human Chemokine standard	2

Table S8 GO-Term enrichment analysis of upregulated genes

p-value adjusted using Bonferroni step-down

GO-Term ID	GO-Term name	% Associated genes	p-value	adj. p-value
GO:0004252	serine-type endopeptidase activity	13.91	4.10E-05	2.11E-02
GO:0005102	signaling receptor binding	11.03	3.27E-09	1.73E-06
GO:0005125	cytokine activity	17.12	9.39E-08	4.94E-05
GO:0005126	cytokine receptor binding	14.83	6.75E-06	3.52E-03
GO:0005509	calcium ion binding	11.21	4.55E-05	2.34E-02
GO:0005539	glycosaminoglycan binding	15.12	1.13E-05	5.87E-03
GO:0008009	chemokine activity	28.85	2.80E-06	1.46E-03
GO:0008083	growth factor activity	17.34	7.69E-06	4.00E-03
GO:0008201	heparin binding	16.67	1.25E-05	6.49E-03
GO:0008236	serine-type peptidase activity	13.35	8.70E-05	4.45E-02
GO:0017171	serine hydrolase activity	13.80	2.67E-05	1.38E-02
GO:0019838	growth factor binding	17.73	2.92E-05	1.51E-02
GO:0030020	extracellular matrix structural constituent conferring tensile strength	36.59	8.50E-08	4.48E-05
GO:0030545	signaling receptor regulator activity	13.18	2.40E-07	1.25E-04
GO:0030546	signaling receptor activator activity	13.65	1.19E-07	6.22E-05
GO:0045236	CXCR chemokine receptor binding	55.56	1.05E-07	5.54E-05
GO:0048018	receptor ligand activity	13.86	6.46E-08	3.41E-05
GO:0048407	platelet-derived growth factor binding	81.82	2.75E-09	1.46E-06
GO:0099094	ligand-gated cation channel activity	15.12	8.59E-05	4.41E-02

Table S9 GO-Term enrichment analysis of upregulated proteins

p-value adjusted using Bonferroni step-down

GO-Term ID	GO-Term name	% Associated genes	p-value	adj. p-value
GO:0008093	cytoskeletal anchor activity	12.50	2.57E-04	7.70E-04
GO:0017166	vinculin binding	21.43	4.79E-05	2.40E-04
GO:0019838	growth factor binding	5.67	7.27E-07	5.09E-06
GO:0030145	manganese ion binding	4.05	6.81E-03	6.81E-03
GO:0045295	gamma-catenin binding	16.67	1.06E-04	4.23E-04
GO:0048156	tau protein binding	6.25	2.00E-03	4.01E-03
GO:0050431	transforming growth factor beta binding	14.81	1.11E-05	6.66E-05

Table S10 GO-Term enrichment analysis of downregulated genes

p-value adjusted using Bonferroni step-down

GO-Term ID	GO-Term name	% Associated genes	p-value	adj. p-value
GO:0000079	regulation of cyclin-dependent protein serine/threonine kinase activity	11.57	1.92E-04	4.34E-02
GO:0000166	nucleotide binding	5.75	8.46E-08	2.22E-05
GO:0000217	DNA secondary structure binding	32.43	6.00E-09	1.62E-06
GO:0000400	four-way junction DNA binding	35.29	2.47E-05	5.78E-03
GO:0003677	DNA binding	5.08	1.23E-04	2.84E-02
GO:0003678	DNA helicase activity	24.71	4.42E-12	1.23E-09
GO:0003684	damaged DNA binding	14.29	1.43E-04	3.28E-02
GO:0003688	DNA replication origin binding	38.10	5.25E-07	1.34E-04
GO:0003697	single-stranded DNA binding	16.91	1.51E-09	4.11E-07
GO:0003887	DNA-directed DNA polymerase activity	35.00	9.94E-11	2.73E-08
GO:0003896	DNA primase activity	85.71	1.93E-08	5.16E-06
GO:0004386	helicase activity	12.43	9.54E-07	2.37E-04
GO:0004672	protein kinase activity	7.15	3.77E-09	1.02E-06
GO:0004674	protein serine/threonine kinase activity	7.24	1.07E-06	2.64E-04
GO:0004693	cyclin-dependent protein serine/threonine kinase activity	10.67	1.80E-04	4.09E-02
GO:0005524	ATP binding	6.35	1.80E-07	4.65E-05
GO:0008017	microtubule binding	13.85	5.89E-13	1.64E-10
GO:0008092	cytoskeletal protein binding	7.02	1.82E-07	4.70E-05
GO:0008094	ATP-dependent activity, acting on DNA	16.90	6.74E-10	1.84E-07

GO:0010997	anaphase-promoting complex binding	66.67	2.17E-07	5.58E-05
GO:0015631	tubulin binding	10.76	4.40E-10	1.21E-07
GO:0016301	kinase activity	6.57	3.37E-08	8.97E-06
GO:0016462	pyrophosphatase activity	6.03	1.29E-05	3.11E-03
GO:0016772	transferase activity, transferring phosphorus-containing groups	6.90	6.27E-11	1.73E-08
GO:0016773	phosphotransferase activity, alcohol group as acceptor	6.58	1.26E-07	3.27E-05
GO:0016779	nucleotidyltransferase activity	10.40	2.77E-05	6.42E-03
GO:0016817	hydrolase activity, acting on acid anhydrides	6.01	1.40E-05	3.34E-03
GO:0016818	hydrolase activity, acting on acid anhydrides, in phosphorus-containing anhydrides	6.01	1.40E-05	3.34E-03
GO:0016887	ATP hydrolysis activity	9.49	5.98E-07	1.51E-04
GO:0016888	endodeoxyribonuclease activity, producing 5'-phosphomonoesters	33.33	1.66E-04	3.79E-02
GO:0017076	purine nucleotide binding	5.72	7.32E-07	1.84E-04
GO:0017108	5'-flap endonuclease activity	62.50	3.87E-06	9.48E-04
GO:0017111	nucleoside-triphosphatase activity	6.06	1.55E-05	3.67E-03
GO:0017116	single-stranded DNA helicase activity	56.00	3.23E-14	9.08E-12
GO:0030554	adenyl nucleotide binding	6.35	8.40E-08	2.21E-05
GO:0032147	activation of protein kinase activity	10.18	1.65E-05	3.89E-03
GO:0032553	ribonucleotide binding	5.70	8.65E-07	2.16E-04
GO:0032555	purine ribonucleotide binding	5.71	8.89E-07	2.21E-04
GO:0032559	adenyl ribonucleotide binding	6.40	5.81E-08	1.54E-05
GO:0034061	DNA polymerase activity	15.74	5.97E-07	1.51E-04

GO:0035173	histone kinase activity	33.33	3.58E-05	8.28E-03
GO:0035639	purine ribonucleoside triphosphate binding	5.56	7.60E-06	1.85E-03
GO:0043085	positive regulation of catalytic activity	6.12	1.36E-05	3.25E-03
GO:0043138	3'-5' DNA helicase activity	37.50	1.65E-05	3.88E-03
GO:0043168	anion binding	5.31	6.66E-06	1.63E-03
GO:0043549	regulation of kinase activity	7.37	3.74E-07	9.57E-05
GO:0045859	regulation of protein kinase activity	7.64	5.83E-07	1.48E-04
GO:0048256	flap endonuclease activity	55.56	8.44E-06	2.04E-03
GO:0051338	regulation of transferase activity	8.48	3.27E-12	9.09E-10
GO:0051347	positive regulation of transferase activity	8.59	1.18E-08	3.17E-06
GO:0071900	regulation of protein serine/threonine kinase activity	8.11	2.56E-05	5.96E-03
GO:0140097	catalytic activity, acting on DNA	13.13	5.42E-13	1.52E-10
GO:1900262	regulation of DNA-directed DNA polymerase activity	57.14	9.81E-09	2.64E-06
GO:1900264	positive regulation of DNA-directed DNA polymerase activity	57.14	9.81E-09	2.64E-06
GO:1901265	nucleoside phosphate binding	5.75	8.67E-08	2.26E-05
GO:1903932	regulation of DNA primase activity	100.00	7.60E-08	2.01E-05
GO:1903934	positive regulation of DNA primase activity	100.00	7.60E-08	2.01E-05
GO:1904666	regulation of ubiquitin protein ligase activity	26.67	1.12E-05	2.69E-03

GO:1904668	positive regulation of ubiquitin protein ligase activity	50.00	2.17E-06	5.33E-04

Table S11 GO-Term enrichment analysis of downregulated proteins

p-value adjusted using Bonferroni step-down

GO-Term ID	GO-Term name	% Associated genes	p-value	adj. p-value
GO:0003678	DNA helicase activity	8.24	5.25E-06	3.67E-05
GO:0003688	DNA replication origin binding	14.29	5.96E-04	1.19E-03
GO:0003697	single-stranded DNA binding	5.15	1.11E-04	4.43E-04
GO:0004386	helicase activity	5.08	1.26E-05	6.30E-05
GO:0008094	ATP-dependent activity, acting on DNA	6.34	2.09E-06	1.67E-05
GO:0016796	exonuclease activity, active with either ribo- or deoxyribonucleic acids and producing 5'-phosphomonoesters	4.62	1.52E-02	1.52E-02
GO:0016887	ATP hydrolysis activity	4.34	4.38E-08	4.38E-07
GO:0017116	single-stranded DNA helicase activity	24.00	3.65E-08	4.01E-07
GO:0035173	histone kinase activity	16.67	3.72E-04	1.12E-03
GO:0043138	3'-5' DNA helicase activity	25.00	6.58E-06	3.95E-05
GO:0140097	catalytic activity, acting on DNA	4.18	4.99E-07	4.50E-06

Table S12 AUC-log2FCs of cytokine secretion during dabrafenib withdrawal

log2FC = log2($AUC_{dabrafenib\ wd}$ +1)-log2($AUC_{DMSO\ wd}$+1). AUC = area under the curve, wd = withdrawal

**cytokine**	**AUC log2FC**
CCL11	5.52
CX3CL1	3.80
CCL2	3.68
CCL7	2.38
IL1b	2.15
CCL17	1.81
LIF	1.51
CXCL10	1.14
CXCL2	1.10
TNF	1.08
CXCL5	1.00
CXCL6	0.83
IL8	0.59
CXCL1	0.47
IL9	-1.08
IL1a	-2.59
